# Pathogenesis and intervention strategies for metabolic dysfunction–associated fatty liver disease from the perspective of the gut–microbiota–liver axis

**DOI:** 10.3389/fimmu.2026.1667180

**Published:** 2026-02-04

**Authors:** Jiabao Liao, Ze Zhou, You Lv, Yiting Zhang, Siyi Liu, Haixia Tang, Fei Qv, Si Wang, Lianhao Yang, Yanming Lu, Zhixia Yang, Xuehua Xie, Mengqiu Shao

**Affiliations:** 1 The First Clinical Medical College, Yunnan University of Chinese Medicine, Kunming, Yunnan, China; 2 Medical Affairs Department, Jiaxing Hospital of Traditional Chinese Medicine, Jiaxing, China; 3 Department of Endocrinology, The First Affiliated Hospital of Yunnan University of Chinese Medicine, Kunming, China

**Keywords:** gut microbiota, gut-microbiota-liver axis, metabolic dysfunction-associated fatty liver disease, microbiota metabolites, microbiota-targeted therapy

## Abstract

Trillions of microorganisms in the human gut are important regulators of health, and the gut and liver have a symbiotic relationship with them. The study found that there is bidirectional communication of substances and signals between the gut and liver, and the gut microbiota is an important medium for mediating bidirectional communication in the gut-liver axis. During metabolic dysfunction-associated fatty liver disease (MAFLD) development, the gut microbiota and its metabolites change to different degrees and affect MAFLD pathogenesis through the gut-liver axis. However, the bidirectional communication mechanism between the gut and liver in MAFLD remains unexplored, and further investigation in this domain is warranted. In this review, we summarize the role of the gut-liver axis in the pathogenesis of MAFLD and explore potential therapeutic strategies targeting intestinal microecology (such as probiotic intervention and phage therapy) to provide a theoretical basis for the precise prevention and treatment of MAFLD.

## 1 Introduction

Metabolic dysfunction-associated fatty liver disease (MAFLD) is the most common chronic liver disease worldwide, with a prevalence rate of 32.4%, which is expected to exceed 55% (1, 2) by 2040. Its disease spectrum covers the gradual progression from metabolic dysfunction-associated steatohepatitis (MASH) to fibrosis and liver cancer (3). Insulin resistance and lipotoxicity are traditionally considered the core drivers of MAFLD (4). However, recent studies have revealed that intestinal microecological disorders play a key role in the gut-microbiota-liver axis, forming a new paradigm for pathological mechanism research (5). The gut and liver interact via portal circulation and bile acid (BA) metabolism, and changes in the structure and function of the gut microbiota have a decisive impact on the liver microenvironment. Clinical and animal model studies have shown that patients with MAFLD exhibit characteristic microbiota dysregulation, increased abundance of proinflammatory bacteria (such as Proteobacteria), significantly reduced barrier-protective bacteria (such as Akkermansia muciniphila), and an abnormal Firmicutes/Bacteroides (F/B) ratio, leading to secondary BA synthesis disorders (6, 7). This imbalance in the gut microbiota leads to multiple pathological effects. Lipopolysaccharide (LPS) cleaved by gram-negative bacteria activates an intrahepatic inflammatory storm via the TLR4/NF-κB pathway. Moreover, short-chain fatty acid (SCFAs) depletion weakens the gut barrier function, resulting in metabolic endotoxemia (8). Notably, microbe-derived trimethylamine N-oxide (TMAO) exacerbates cholestasis by inhibiting farnesoid X receptor (FXR) signaling, revealing that microbial metabolites have dual inflammatory and metabolic regulatory effects in MAFLD (9, 10).

Although the gut-microbiota-liver axis theory provides a new perspective on the mechanism of MAFLD, some key scientific questions regarding its multidimensional interactions remain unanswered. First, the heterogeneity of the gut microbiota leads to insufficient reproducibility of clinical study results, making it challenging to understand their impact. Second, the concentration-dependent effect thresholds for metabolites, such as BAs and SCFAs, have not been defined, which limits our understanding of their mechanisms of action. Furthermore, the lack of a systematic interpretation of the spatiotemporal dynamics of interactions between the microbiota and host within immune microenvironments adds an additional layer of complexity to such studies. In this review, we systematically describe how gut microbiota drives MAFLD progression through the exchange of metabolites, immune signal transmission, and epigenetic regulation, integrating the structure and function framework of the gut-microbiota-liver axis. Based on this, we propose a refined treatment strategy for targeted microbiota intervention, thereby establishing a theoretical foundation for overcoming the bottleneck in the prevention and treatment of MAFLD. Importantly, beyond the classical gut–liver axis, emerging evidence supports a gut–spleen–liver immune circuit that contributes to sustained low-grade inflammation and systemic immune dysregulation during MAFLD/MASLD progression. Accordingly, while this review is structured around gut microbiota–derived metabolic and barrier signals, we incorporate the spleen as a peripheral immune “amplifier/hub” to better explain the coupling between systemic cytokine tone and the hepatic inflammatory microenvironment.

## 2 Anatomical and physiological basis of the gut-microbiota-liver axis

### 2.1 Bidirectional regulation of the gut-liver axis

The functional synergy between the liver and gut is rooted in their embryonic homology. The embryonic foregut endoderm differentiates into hepatic progenitor cells and the intestinal epithelium, which is the basis of their anatomical and functional interactions through the portal vein (transporting intestinal metabolites) and the biliary system (secreting BAs and immune factors) (11). The liver relies on the portal venous system for approximately 75% of its blood supply, making it the primary “sensor” (12) of intestinal metabolite levels. The liver actively regulates the composition of the gut microbiota through the secretion of IgA and antibacterial peptides by the biliary tract, forming a bidirectional regulatory circuit that links metabolism and immunity (13). In addition, primary BAs synthesized by the liver (cholic acid [CA] and chenodeoxycholic acid [CDCA]) are converted into secondary BAs (deoxycholic acid [DCA] and lithocholic acid [LCA]) by the gut microbiota (such as Bacteroides and Clostridium). Approximately 95% of these are reabsorbed through enterohepatic circulation (14). Secondary BAs activate FXR and G protein-coupled BA receptor 1 (TGR5), regulate glycolipid metabolism, and inhibit inflammation (14). This process presents a characteristic disorder in MAFLD: dysregulation of the microbiota leads to reduced secondary BAs production, impaired FXR-mediated lipid oxidation, and increased hepatic fat deposition. The effects of gut microbiota on the host are a double-edged sword. SCFAs (e.g., butyric acid) enhance gut barrier integrity and inhibit hepatic steatosis by activating GPR41/43 receptors (15). Conversely, excess LPS activates the Toll-like receptor 4 (TLR4) pathway in hepatic Kupffer cells via portal vein translocation, driving insulin resistance and fibrosis progression (16). This fine regulation of the microbial-host metabolic interface highlights the pivotal role of the gut-liver axis in disease development. To avoid conceptual overlap, we operationally distinguish the “gut–liver axis” from the “gut–spleen–liver axes.” Here, the gut–liver axis primarily refers to the portal delivery of microbial components and metabolites (e.g., LPS, bacterial DNA, SCFAs, and bile acid intermediates) following dysbiosis and barrier disruption, which directly triggers hepatic innate immune and metabolic responses, while liver-derived bile acid and immune signals reciprocally reshape the gut ecosystem. In contrast, the gut–spleen–liver axis emphasizes immune amplification. The spleen integrates gut-derived antigenic and inflammatory cues, modulates T-cell polarization and monocyte/macrophage activation, and increases cytokine output. Splenic immune cells can subsequently migrate to the liver and reinforce the inflammatory and profibrotic milieu. Together, these pathways form a triangular immunometabolic network with partially overlapping inputs, but distinct dominant drivers and readouts.

### 2.2 Physiological barrier system of the gut-microbiota-liver axis

As the core interface separating the gut microbiota from the host’s internal environment, the intestinal mucosal barrier maintains a balance in microbiota-host interactions through four defense mechanisms (mechanical, biological, chemical, and immune), and its functional integrity directly affects liver metabolism and immune homeostasis.

#### 2.2.1 Mechanical barriers: dynamic regulation of tight junction proteins

The mechanical barrier, located beneath the mucus layer, is the most important factor affecting the selective permeability of the intestinal mucosa (17, 18). This barrier is composed of specialized epithelial cells, including goblet, Paneth, and M cells. The apical junction complex (AJC) is a critical structural component that consists of TJs and adherens junctions (AJs), the two primary modes of cellular connection (19, 20). TJ, the core regulatory element of the mechanical barrier, are composed of transmembrane proteins (claudins, occludin, and junctional adhesion molecules) and cytoplasmic scaffold proteins (ZO-1/2/3), which maintain barrier integrity through dynamic anchoring to the cytoskeleton (21–23). ZO-1 plays a central regulatory role in maintaining the structural stability of AJC through its interaction with cytoskeletal proteins (24). Studies have demonstrated that TJ protein expression levels are significantly negatively correlated with intestinal permeability, and that ZO-1 is an established biomarker for assessing TJ function (25). Under pathological conditions, the expression of ZO-1 is downregulated, resulting in endotoxin (such as LPS) translocation and activation of the TLR4/NF-κB pathway in liver Kupffer cells, driving liver inflammation and insulin resistance (26, 27).

#### 2.2.2 Biological barriers: bi-directional regulation of microbiota metabolites

Bacteroidetes and Firmicutes are the dominant phyla in the human gut microbiota. Actinobacteria and Proteobacteria cooperate to form multidimensional biological barrier (28). These symbiotic bacteria prevent the colonization of pathogenic bacteria, such as Salmonella, by secreting bacteriocins, producing SCFAs, and competitively deprressing pathogenic bacteria of essential nutrients. Butyric acid not only decreases intestinal pH and inhibits pathogen proliferation, but also activates the peroxisome proliferator-activated receptor gamma (PPARγ) signaling pathway in the intestinal epithelium, upregulates TJ protein expression, and strengthens the mechanical barrier (29). The gut microbiota also maintains immune tolerance to symbiotic bacteria by inducing regulatory T cell (Treg) differentiation and secretory IgA (sIgA) production through low-intensity interactions of model molecules (such as LPS and peptidoglycan) with intestinal epithelial TLR. Notably, Kupffer cells clear portal vein microbiota antigens and block systemic inflammation, while hepatic-originated complement components, such as complement component 5a (C5a), exert feedback regulation on the secretion of intestinal sIgA, forming closed-loop immune homeostasis (30, 31). In contrast, hepatic primary BAs (CA and CDCA) are transformed into secondary BAs (DCA and LCA) by the gut microbiota (e.g., Bacteroides and Clostridium), which enhance barrier function through the following mechanisms: 1) direct antibacterial effect (hydrophobic BAs destroy pathogen membrane structure) (32) and 2) signal activation (conjugated BAs activate intestinal epithelium FXR, promote antibacterial peptide [e.g., ANG4] expression, and mucus secretion) (33). Additionally, microbiota metabolites, such as tryptophan derivatives, are transported to the liver via the portal vein, regulating the FXR/FGF19 signaling axis, inhibiting lipid synthesis, and maintaining BA homeostasis (34).

#### 2.2.3 Chemical barriers: coordinated regulation of BAs and digestive enzymes

The intestinal chemical defense system forms a cascade of antibacterial barriers through the synergistic action of gastric acid, BAs, digestive enzymes (including proteases and lipases), lysozymes, and antimicrobial peptides (AMPs) (35). Among them, gastric acid, trypsin, and lysozyme play a non-specific bactericidal role by destroying microbial cell walls (such as the outer membrane of gram-negative bacteria) or degrading antigenic proteins, thus maintaining the integrity and function of the gut barrier (36). BAs play a dual regulatory role in this system (37). On one hand, they act as physiological detergents, with hydrophobic BAs (e.g., DCA) disrupting the membrane structures of gram-positive bacteria at critical concentrations (> 2 mM). On the other hand, they act as signaling molecules, with conjugated BAs activating FXR/TGR5 receptors to upregulate TJ protein (occludin) expression and drive microbiota-mediated secondary BA conversion. In addition, AMPs (e.g., defensins and cathelicidins) disrupt pathogen membrane integrity through charge interactions while exhibiting selective tolerance to symbiotic bacteria, thereby maintaining barrier homeostasis (38).

#### 2.2.4 Immune barriers: sIgA immune dialogue with the liver

**Figure 1:**
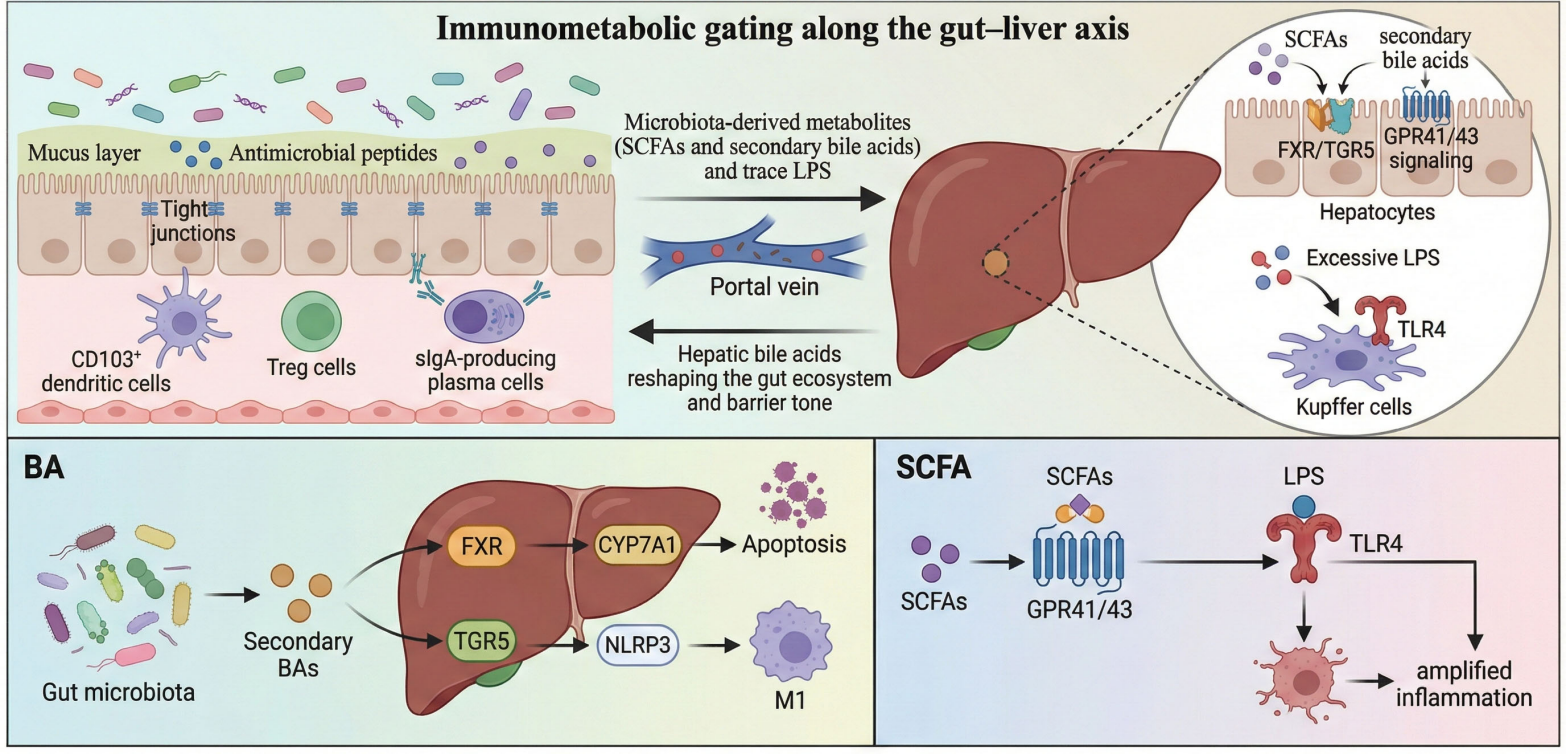
Immunometabolic gating along the gut–liver axis. The gut–liver axis is a bidirectional circuit that integrates intestinal barrier immunity and hepatic innate sensing. Mucus, antimicrobial peptides, and epithelial tight junctions limit microbial translocation, whereas CD103^+^ dendritic cells, Treg cells, and sIgA-producing plasma cells maintain mucosal tolerance. Microbiota-derived metabolites (SCFAs and secondary bile acids) and trace LPS reach the liver via the portal vein to modulate FXR/TGR5 and GPR41/43 signaling, whereas excessive LPS activates TLR4 in Kupffer cells to amplify inflammation. Hepatic bile acids return to the gut, reshaping the gut ecosystem and barrier tone. SCFAs, short-chain fatty acids; LPS, lipopolysaccharide; sIgA, secretory immunoglobulin A; FXR, farnesoid X receptor; TGR5, bile acid receptor; GPR41/43, SCFA receptors; TLR4, Toll-like receptor 4.

The gut immune barrier is primarily composed of gut-associated lymphoid tissue (GALT) and sIgA. Among them, GALT sustains mucosal immune homeostasis through the “antigen sampling-immune education-effect output” cascade. The core mechanism is that micropleated cells (M cells) transport intestinal lumen antigens to Pyle’s node, and integrin alpha E (CD103)^+^ dendritic cells induce Treg differentiation through retinoic acid signaling, thus establishing immune tolerance to symbiotic bacteria (39). Furthermore, the gut-specific chemokine chemokine (C-C motif) ligand 25 (CCL25) directs plasmablasts to migrate to the lamina propria and differentiate into sIgA plasma cells, which produce 80% of intestinal immunoglobulins (40). Subsequently, sIgAs block pathogen adhesion and invasion by binding to fragment antigen-binding (Fab) fragments (such as flagellin). In addition, sIgA can limit the excessive proliferation of symbiotic bacteria through Fc alpha receptor (FcαR)-mediated encapsulation and maintain the spatial colonization stability of the microbiota (41). Clinical studies have shown that reduced sIgA levels lead to abnormal amplification and translocation of proteobacteria, such as Escherichia coli, which activate the TLR4/NF -κB pathway in Kupffer cells via the portal vein, driving hepatic inflammation and metabolic disorders (42). In addition to sIgA, intestinal epithelial cells release IgE, IgG, and various other immunoglobulins that play key roles in maintaining intestinal humoral immunity. IgG activates neutrophil extracellular trap formation (NETosis) via FcγR in areas of gut barrier injury, thereby exacerbating tissue inflammation (43) (**Table 1**). In summary, the gut–liver axis is a bidirectional metabolic–immune circuit primarily mediated by portal circulation and enterohepatic bile acid cycling. Its physiological “gatekeeping” relies on multilayer intestinal barriers (mechanical, biological, chemical, and immune) that constrain microbial translocation while preserving host–microbiota mutualism. Disruption of these barriers and bile acid/SCFA signaling shifts the hepatic microenvironment toward steatosis-prone metabolism and inflammation, providing a structural basis for MAFLD initiation and progression (**Figure 1**). Building on this barrier–mucosal immune framework, the spleen can function as a key “gain-control” organ in systemic immunity. When gut-derived antigenic load and inflammatory cues increase, splenic immune programming (e.g., T-cell polarization and monocyte/macrophage activation) can elevate the basal inflammatory tone and cytokine output. Consequently, splenic immune cells may be recruited to the liver, amplifying intrahepatic inflammation and fibrogenic signaling. This cross-organ circuitry supports the view that immune dysregulation in MAFLD/MASLD is not purely liver-centric but can emerge from coordinated gut–spleen–liver crosstalk.

**Table 1:** Barrier system from the gut-liver axis perspective: structure-function integration and pathological mechanism.

Barrier type	Core components and key molecules	Functional mechanism	Pathological association
Mechanical barrier	Specialized epithelial cells (goblet cells, Paneth cells, M cells) (19, 20); apical junction complex (AJC) tight junction (TJ) proteins (claudins, occluding, JAMs, ZO-1/2/3) (21, 23)	The AJC connects epithelial cells through TJ and AJ. ZO-1 anchors the cytoskeleton to maintain barrier Integrity (24). TJ protein expression is negatively correlated with intestinal permeability ZO-1 is a core regulatory protein (25).	ZO-1 downregulation leads to increased intestinal permeability, LPS translocation, activation of TLR4/NF-κB pathway in hepatic Kupffer cells, Liver inflammation, and insulin resistance (26, 27).
Biological barrier	Gut microbiota (e.g., Bacteroides and Firmicutes) (28); short-chain fatty acids (SCFAs) (e.g., butyric acid) and secondary bile acids (BAs) (e.g., DCA and LCA) (29)	SCFAs secreted by microbiota activate peroxisome proliferator-activated receptor gamma (PPARγ) in the intestinal epithelium and upregulate TJ proteins (30, 31). Secondary BAs destroy pathogen membrane structure and activate farnesoid X receptor (FXR), enhancing antimicrobial peptide expression and microbiota -liver interaction (32). Tryptophan metabolites regulate the FXR/FGF19 axis to maintain BA homeostasis (33).	Microflora imbalance and LPS translocation cause systemic inflammation (30, 31), abnormal BA conversion, and weakened antibacterial function (34). Gut-liver axis disorders exacerbatemetabolic disease (44).
Chemical barrier	BAs (CA, CDCA, and DCA) (35), digestive enzymes(proteases, lipases, lysozymes, and antimicrobial peptides (AMPs)[e.g., defensins) (36)	Hydrophobic BAs (> 2 mM) disrupt pathogen membranes (38). Digestive enzymes degrade microbial antigens. AMPs selectively kill pathogens and maintain microbiotabalance (38) FXR/TGR5 activation enhances occludin expression (38).	Abnormal BA concentrations, increased risk of Gram-positive infections38, inadequate AMPs, pathogen proliferation, digestive enzyme deficiencies, antigen clearance disorders, and barrier disruption (38)
Immunologic barrier	gut-associated lymphoid tissue (GALT) (with M cells and Treg), secretory immunoglobulin A(sIgA) (39), chemokine Chemokine (C-C motif) Ligand 25 (CCL25) chemokine, and IgG/IgE (41).	M cells present antigens, Treg differentiation induces immune tolerance (40). CCL25 induces plasma cells to produce sIgA, blocks pathogen adhesion, and limits microbiota hyperproliferation (41). Hepatic Kupffer cells clear antigen, and Complement component 5a (C5a) feedback regulates sIgA secretion (42).	sIgA levels decreased Proteota amplification, bacterial translocation, and activation of the hepatic TLR4/NF-κB pathway (43). IgG activates neutrophil extracellular trap formation (NETosis) and increases inflammation in damaged areas (45).

## 3 Alterations in gut microbiota composition in MAFLD patients

**Figure 2:**
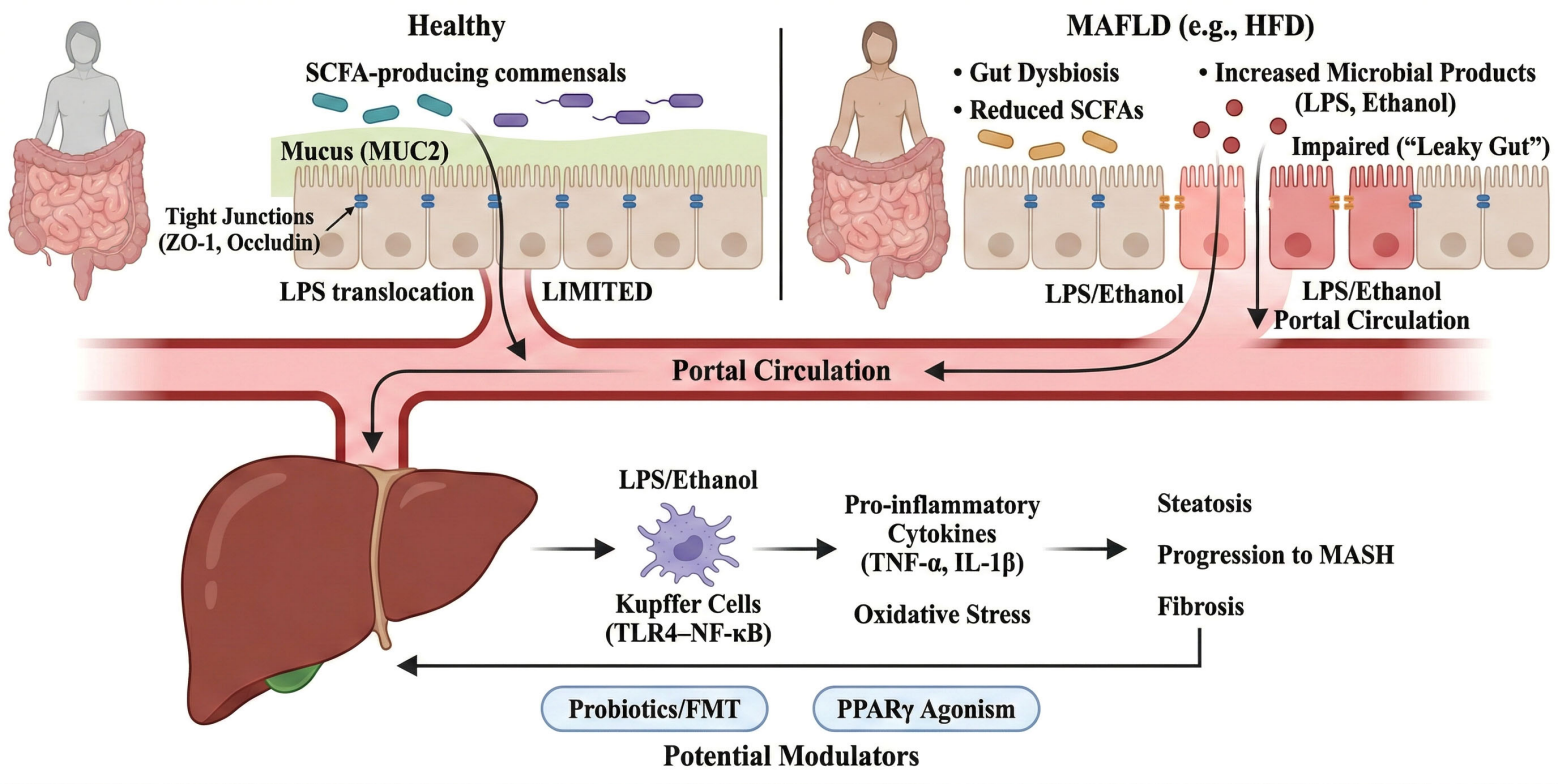
Gut dysbiosis–barrier dysfunction axis drives MAFLD progression via portal circulation. In healthy individuals, SCFA-producing commensals support mucus (MUC2) and tight junction integrity (e.g., ZO-1 and occludin), limiting luminal LPS translocation. Under MAFLD-associated dysbiosis (e.g., a high-fat diet), reduced SCFAs and increased microbial products (LPS and ethanol) impair barrier function and increase permeability (“leaky gut”). Portal delivery of LPS/ethanol activates Kupffer cells via TLR4–NF-κB, inducing pro-inflammatory cytokines (e.g., TNF-α, IL-1β) and oxidative stress, thereby driving steatosis, progression to MASH and fibrosis. Probiotics/FMT and PPARγ agonism are shown as potential modulators. MAFLD, metabolic dysfunction–associated fatty liver disease; MASH, metabolic dysfunction–associated steatohepatitis; SCFAs, short-chain fatty acids; FMT, fecal microbiota transplantation; HFD, high-fat diet; LPS, lipopolysaccharide; TJ, tight junction; ZO-1, zonula occludens-1.

The gastrointestinal tract contains hundreds of bacteria that are intimately involved in the physiological functions of the body through metabolites, immune regulation, microbial components and neuromodulation. The gut microbiota plays a crucial role in metabolism, genetics, and immune regulation of the host. To date, studies have found that changes and imbalances in the gut microbiota are strongly associated with acute pancreatitis, intestinal diseases, cardiovascular diseases and cancers. In recent years, numerous studies have demonstrated that gut microbiota plays a role in the pathogenesis of MAFLD, and alterations in gut microbiota have been observed in both patients and animal models of MAFLD. We provide an overview of the gut microbiota in MAFLD, laying the foundation for further exploration of the role and mechanisms of gut microbiota in this condition (**Figure 2**).

### 3.1 Patients with MAFLD

Recent studies have found that patients with MAFLD have characteristic disorders in their gut microbiota. Multiple cohort studies have shown that, compared with healthy individuals, patients with MAFLD have an increased abundance of Bacteroidetes and a decreased abundance of Firmicutes, with SCFA-producing Lachnospiraceae and Lactobacillaceae being significantly reduced (46, 47). Proteobacteria and its subordinate Enterobacteriaceae, especially ϵ-proteobacteria and γ-proteobacteria, abnormally proliferated in children with MAFLD. The microbiota characteristics differed significantly at different stages of the disease. Firmicutes and Eubacterium rectale were dominant in patients with mild/moderate MAFLD, whereas Proteobacteria abundance increased in patients with advanced fibrosis, especially Escherichia coli proliferation (48, 49). Notably, ethanol-producing microbiota (e.g., Escherichia spp. of the Enterobacteriaceae family) are specifically elevated in patients with MASH, and their abundance is positively correlated with serum ethanol concentration (50, 51). A random forest model analysis revealed that the diagnostic model based on marker bacteria, such as Ruminococcus and Enterococcus, exhibited excellent discrimination ability for advanced fibrosis (52). A study also revealed population heterogeneity in microbiota changes, with Prevotella hyperproliferation in obese children with MAFLD and abnormal increases in Propionibacterium and Parabacteroides in adults (53). Notably, baseline Erysipelothrix levels were positively correlated with the risk of hepatic fat accumulation, whereas Gammaproteobacteria had a protective effect (54). These microbiota characteristics were significantly associated with clinical parameters, such as gut-derived LPS levels and liver inflammation, suggesting that microbiota-host interactions play a key role in MAFLD progression.

### 3.2 Animal models with MAFLD

In rodent models of MAFLD, gut microbiota disturbance is frequently associated with liver pathology. High-fat diet (HFD)-induced MAFLD mice exhibited characteristic Changes in gut microbiota composition, including a marked increase in the abundance of Firmicutes and Proteobacteria, as well as a notable decrease in the abundance of Bacteroidetes and beneficial bacteria with immune regulatory functions (such as Bifidobacterium) (55, 56). In these models, dysbiosis is accompanied by impaired gut barrier function, manifested by reduced secretion of mucin 2 (MUC2), downregulation of TJ proteins (occludin and ZO-1), and increased intestinal permeability, which can increase the translocation of endotoxins (LPS) into the bloodstream and is linked to systemic inflammation (57). Mechanistic evidence from animal experiments suggests that HFD decreases the production of antimicrobial peptides (e.g., Reg3-γ) and mucins by inhibiting the PPARγ signaling pathway, whereas the PPARγ agonist rosiglitazone has been shown to restore gut barrier function (58, 59). In addition, fecal microbiota transplantation (FMT) studies in mice provide causal support within the experimental setting: recipient mice develop typical MAFLD-like phenotypes, such as steatosis, inflammatory infiltration, and insulin resistance, after germ-free mice are colonized with gut microbiota from MAFLD donors, supporting a causal contribution of microbiota configurations in this model rather than establishing causality in humans (6). Notably, the absence of Bifidobacterium is associated with increased liver inflammation. However, its protective effects may involve metabolism-independent pathways, such as Toll-like Receptor 2 (TLR2)-mediated immune modulation. In experimental models, Proteobacteria proliferation-driven activation of the LPS–TLR4/NF-κB pathway has been proposed to further exacerbate hepatocyte damage (60). These findings collectively support the notion that, in animal models, the gut microbiota can modulate MAFLD-related phenotypes through the “microbiome–gut–liver axis” and provide an experimental basis for intervention strategies targeting the microbiota (e.g., probiotics and FMT) or the PPARγ pathway. Taken together, both clinical cohorts and animal models indicate that MAFLD is accompanied by stage-dependent dysbiosis. Importantly, transplantation and perturbation studies in rodents support causality within the model, whereas confirmation in humans requires adequately powered interventional trials.

## 4 Microbiota risk factors for MAFLD

### 4.1 Exogenous risk factors

#### 4.1.1 Diet and nutrition

Nutrition and dietary composition are key factors regulating the structure and function of gastrointestinal microbiota communities. Early cross-regional studies revealed that African children following a traditional high-fiber diet exhibited a 38% Prevotella/Enterobacteriaceae ratio. In contrast, Italian children on a Westernized diet had a higher Firmicutes/Enterobacteriaceae ratio, which was closely associated with the conversion of excess choline into trimethylamine (TMA) catalyzed by choline trimethylamine-lyase (CutC) and its activator CutD in Western diets and the subsequent production of Trimethylamine N-oxide (TMAO), a proinflammatory mediator. A modern refined diet (fiber < 15 g/day) decreases Prevotella abundance and SCFA production, directly impairing PPARα-mediated fatty acid oxidation (61). Similarly, a low-fiber diet reduces microbiota BA hydrolase activity, leading to an accumulation of primary BAs(e.g., CA), which inhibits Cytochrome P450 family 7 subfamily A member 1 (CYP7A1) expression by activating the FXR/SHP pathway in hepatocytes and exacerbating cholesterol metabolism imbalance (62, 63). HFD has profound effects on the composition of the gut microbiota, and remodeling of the gut microbiota occurs independently of obesity. In mice fed HFD for 8 weeks, the abundance of Proteobacteria increased, and that of SCFA-producing Roseburia decreased (64, 65). In addition, HFD induced the upregulation of microbiota choline metabolic pathway activity, which was accompanied by a simultaneous increase in serum TMAO levels (66). Transplantation of gut microbiota from HFD-fed donor mice into germ-free recipients recapitulated the phenotypes of hepatic steatosis, inflammation, and fibrosis, confirming that microbiota disorder is the core medium of diet-induced liver damage (8). In a cohort study of patients with advanced liver disease, dietary patterns rich in coffee (≥ 3 cups/day), whole grains (> 30%), and fermented dairy products (200 g per day) significantly increased intestinal microbial alpha diversity and reduced the risk of hospitalization (67). In further animal studies, a chronic high-fat high-cholesterol (HFHC) diet (> 14 months) induced liver steatosis (14 weeks), inflammation (20 weeks), fibrosis (36 weeks), and liver cancer (56 weeks) progression. Concurrently, the bile salt hydrolase (BSH) activity of gut microbiota was reduced, resulting in the inhibition of intestinal FXR signaling and intensification of lipid accumulation in the liver. Thus, the evolution of liver steatosis and liver inflammation in MAFLD has evolved into a classic example of diet-induced inflammation, suggesting that excess nutrients can spread disease along the “gut-liver axis.”

#### 4.1.2 Biological clock

The biological clock is the core regulatory system that enables organisms to adapt to day–night environments, and its interaction with the gut microbiota has been gradually clarified. Studies have demonstrated that approximately 20% of the gut microbiota exhibit circadian fluctuations in abundance and metabolic function, and experimental evidence supports that these oscillations are strongly shaped by host behavioral timing (especially feeding schedules), rather than implying a universal direct regulatory mechanism. The gut microbiota prioritizes energy metabolism and DNA replication during the daytime and shifts to detoxification pathways and SOS response-mediated DNA repair at night. Moreover, the feeding rhythms of the host not only drive diurnal reprogramming of microbiota functions but also shape dynamic fluctuations in microbiota composition. Together, these observations suggest that feeding cycles act as major zeitgebers (timing cues) for the microbial rhythmicity (68, 69).

It is worth noting that defects in core clock genes in animal models are associated with an imbalance in microbiota homeostasis. Mutations in the circadian locomotor output cycles kaput (Clock) have been reported to be accompanied by the proliferation of proinflammatory Enterobacteriaceae, and deletions of circadian rhythm target genes Per 1/2 or brain and muscle ARNT-like protein 1 (BMAL1) disrupt microbial rhythmicity and are linked to gut barrier dysfunction (70). These findings indicate that the biological clock system sustains symbiotic equilibrium between the microbiota and host through a dual mechanism involving genetic regulation and environmental cues, such as photoperiod and feeding time. Furthermore, its disruption may contribute to the pathogenesis of metabolic diseases by altering microbial rhythms, although its causal relevance in humans requires interventional confirmation.

In addition, in a germ-free mouse experiment, transplantation of gut microbiota from trans-time travelers was sufficient to induce obesity and impaired glucose tolerance in recipient animals (71, 72), supporting a causal contribution of altered microbiota configurations within this experimental setting. Furthermore, although HFD disrupts the circadian rhythm of the microbiota and induces obesity and metabolic syndrome, limiting the time window of HFD intake (time-restricted feeding in mice) can restore glucose metabolism homeostasis and suppress weight gain (73, 74). This protective effect has been associated with a partial restoration of gut microbiota rhythmicity, manifested by a decrease in the abundance of obesity-associated bacteria and an increase in the functional activity of beneficial taxa, such as Oscillibacter, Ruminococcus, and SCFA-producing strains (75). Further studies have revealed that HFD intake with circadian rhythm disruption in experimental models exacerbates gut microbiota dysregulation, resulting in impaired gut barrier function and endotoxin entry into the bloodstream, which is linked to systemic inflammation and may accelerate metabolic syndrome progression (76). These findings suggest that regulating microbial circadian rhythms is a mechanistically plausible intervention avenue supported by animal studies; however, translation to humans will require well-designed clinical trials with time-resolved microbiome and metabolic endpoints.

#### 4.1.3 Smoking

Smoking, a global public health problem, is closely associated with the onset and progression of MAFLD. Large-scale population-based studies have shown that smoking is an independent risk factor for MAFLD, and its harmful effect are dose-dependent (77, 78). A cross-sectional analysis of 6,852 patients with MAFLD showed that those who smoked ≥ 20 cigarettes per day had a significantly higher risk of liver fibrosis than light smokers (< 10 cigarettes per day), with an increased risk of fibrosis of 30% per 10 years of smoking history. A multicenter cohort study from 16 centers in the United States further confirmed that smokers had a significantly higher risk of liver steatosis, lobular inflammation, and cirrhosis progression over 5 years than nonsmokers (79). In addition, there are sex differences in the effects of smoking on MAFLD. A multicenter cohort study conducted in China demonstrated that female smokers have a 1.7-fold higher risk of developing MASH than male smokers, and that liver fibrosis progresses faster among females (80). Recent studies have revealed that gut microbiota disorders are central to the mechanism by which smoking exacerbates MAFLD. Nicotine and other tobacco smoke components can directly interact with the gut microbiota, remodeling its structure. For example, the abundance of proinflammatory bacteria (such as Prevotella and Veillonella) in the intestinal tract of smokers increases significantly, whereas the proportion of beneficial bacteria, such as Firmicutes, decreases. Inhibition of gut colonization by SCFA-producing bacteria (such as Bifidobacterium) decreases SCFA levels in the cecum and increases intestinal pH, thus destroying the integrity of the gut barrier (81). Smoking also significantly increases the proportion of Gram-negative bacteria such as Proteobacteria, resulting in increased LPS production (82). LPS is transferred to the liver through the portal vein, binds to TLR4 on Kupffer cell surfaces, activates the NF-κB pathway, drives the release of proinflammatory factors such as TNF-α and IL-6, and triggers insulin resistance and excessive lipid deposition in hepatocytes (83, 84). Animal models have provided direct evidence for this mechanism. SCFA levels in the cecum of rats exposed to cigarette smoke for 4 weeks were reduced, and antimicrobial peptide expression was suppressed. Additionally, in the intestines of mice treated with cigarette smoke condensate, the abundance of pathogenic bacteria, such as Erysipelothrix, is increased, accompanied by increased serum LPS levels and increased liver steatosis (85–88). In conclusion, smoking may affect the composition of the gut microbiota by changing the intestinal microenvironment (such as excessive proliferation of pathogenic bacteria), thereby regulating the host immune inflammatory response and SCFA production, ultimately leading to MAFLD progression.

#### 4.1.4 Atmospheric particulates

Particulate matter with a diameter of less than 2.5 micrometers (PM2.5), a major air pollutant in China, has attracted considerable attention owing to its extensive exposure and multi-organ toxicity. Recent studies have found that PM2.5 not only directly damages the respiratory system but also becomes a new environmental driver of MAFLD progression by reshaping intestinal microecology, disrupting the intestinal mucosal barrier, and triggering systemic inflammatory responses. In animal experiments, short-term exposure (7 days) can cause significant spatial heterogeneity in the gastrointestinal microbiota of mice. β-diversity increases from the proximal (stomach) to the distal (colon) regions, suggesting that PM2.5 exerts a stronger selective pressure on the distal intestine (89). Notably, prolonged exposure (12 weeks) did not significantly reduce the α-diversity of the microbiota, but resulted in a decrease in the relative abundance of Firmicutes and a 2-fold increase in Proteobacteria, resulting in the development of a proinflammatory microbiota phenotype (90). Further studies have shown that PM2.5 reduces cecal butyrate levels by inhibiting the growth of SCFA-producing bacteria (such as Roseburia and Faecalibacterium) and impairs its regulatory effect on liver lipid metabolism (91). In addition to dysbiosis, PM2.5 impairs gut barrier integrity via a dual pathway. PM2.5 downregulates the mRNA expression of the TJ protein ZO-1 and occludin in intestinal epithelial cells and induces a burst of mitochondrial reactive oxygen species (ROS) burst (92). ROS-mediated apoptosis: Excessive ROS activate the JNK/c-Jun pathway, triggering apoptosis of colonic epithelial cells and causing a mucosal mechanical barrier breach. These changes cause metabolic endotoxemia-gut microbiota disorder, promote increased LPS production and translocation to the portal venous circulation through the damaged barrier, activate the TLR4/NF-κB pathway in Kupffer cells, and increase the release of proinflammatory factors (TNF-α and IL-6) (93).

#### 4.1.5 Drugs

Proton pump inhibitors (PPIs), the most widely used antacids worldwide, have been associated with gut microbiota disorders in multi-omics studies. A large population cohort study in the Netherlands revealed reduced α-diversities in the gut microbiota of PPI users and an abundance shift (94) in nearly one-fifth of the microbiota taxa. Metagenomic analysis further confirmed that PPIs significantly altered the activity of 133 metabolic pathways involved in the functional remodeling of lipid synthesis, Nicotinamide Adenine Dinucleotide (NAD +) fermentation, and purine degradation. This phenomenon of “oral-intestinal microbiota homology” results in the loss of integrity of the intestinal mechanical barrier and increased translocation of endotoxins (e.g., LPS) (95). The release of LPS into the blood drives the secretion of hepatic inflammatory factors (TNF-α and IL-6) by activating the TLR4/NF-κB pathway in Kupffer cells and inhibiting insulin receptor substrate phosphorylation, thereby exacerbating hepatocyte steatosis and MAFLD progression (96). In addition, PPIs induce microbiota imbalance and barrier damage to form a vicious circle, eventually promoting MAFLD progression to the fibrosis stage. This finding suggests that the risks associated with long-term PPI use should be considered in clinical practice.

Metformin is a first-line Type 2 diabetes mellitus (T2DM) drug, and its hypoglycemic effect is partially achieved through microbiota-host interaction. Intervention studies have shown that metformin specifically increases the abundance of SCFA-producing bacteria while inhibiting inflammation-associated enterobacteriaceae (97). Transplantation experiments conducted in germ-free mice demonstrated that microbiota reshaped by metformin treatment led to a reduction in blood glucose levels and an enhancement in glucagon-like peptide-1(GLP-1) secretion (98). However, this repopulation also resulted in adverse reactions, such as diarrhea/abdominal distension, which may be related to the increased intestinal gas production caused by the hyperproliferation of Methanobrevibacterium (99). Studies suggest that metformin’s modulation of the gut microbiota may have potential benefits for patients with MAFLD by improving gut microbiota structure and reducing inflammation levels, thereby reducing liver steatosis and inflammatory response (100).

### 4.2 Endogenous host factors

#### 4.2.1 Delivery patterns

The traditional “sterile uterus” hypothesis has been overturned, and the latest metagenomic studies have confirmed that human fetal intestinal colonization begins *in utero*. Low biomass but highly specific microbiota communities (such as Lactobacillus and Bifidobacterium) were detected in the placenta and amniotic fluid, with 16S rRNA gene abundance of 10^^2^–10^^3^ copies/g tissue (101). In mouse models, orally administered fluorescently labeled Enterococcus faecium can cross the placental barrier via maternal circulation and colonize the fetal intestine, and the detection rate of labeled bacteria in the meconium microbiota is approximately 70% (102). This finding indicates a potential pathway for vertical maternal-fetal microbial transmission. The mode of delivery is a decisive factor for the initial microbiota of newborns. Lactobacillus crispus and Prevotella bivia from the maternal vagina are the main gut microbiota of newborns, and they are metabolically characterized by SCFAs synthesis pathway enrichment (103). The microbiota may also be derived from maternal skin bacteria (such as Streptococcus and Cutibacteria), which is accompanied by an increase in LPS biosynthesis pathway activity (10). These primary microbiota evolve to become more diverse and relatively stable. At 3 years of age, the gut microbiota of children becomes similar to that of adults. Early colonization and composition of the gut microbiota have profound effects on the host’s metabolic health. Studies have shown that the composition of the early gut microbiota is closely related to the metabolic state of the host, especially with the onset and development of MAFLD (104). For instance, microbiota disruption leads to the downregulation of occludin expression, an increase in intestinal permeability, enhanced endotoxin (LPS) translocation, and activation of the TLR4/NF-κB pathway in hepatic Kupffer cells, thereby promoting TNF-α secretion and hepatic fat deposition (6). In addition, certain bacterial species (e.g., Akkermansia muciniphila) regulate BA metabolism by inducing FXR signaling and inhibiting hepatocyte lipid synthesis, whereas Clostridium-derived secondary BAs enhance white fat browning (105) via the TGR5-cAMP-PKA pathway (106, 107). Thus, colonization and composition of the early gut microbiota may provide new insights into the prevention and treatment of MAFLD.

#### 4.2.2 Aging

The global aging population is accelerating, and the population aged 65 and over is projected to exceed 20% (approximately 1.5 billion) by 2050. Among these, 80% will be in low- and middle-income countries, and the prevalence of MAFLD in this population is expected to increase significantly (108). Aging drives gut-liver axis imbalance by remodeling the structure and function of the gut microbiota. The α-diversity of the gut microbiota in older adults is significantly lower than that in younger individuals and is imbalanced, characterized by the expansion of pathogens and the depletion of symbiotic bacteria (109, 110). This disorder results in a decrease in SCFA synthesis and a concomitant decrease in 7α-dehydroxylase activity, leading to the accumulation of unconjugated BAs (DCA and LCA), which, in turn, inhibits ileal FXR signaling (111). Gut barrier injury increases LPS levels in the portal vein, inhibits insulin receptor phosphorylation through activation of the hepatic TLR4/MyD88 pathway, and stimulates TGF-β1 secretion by hepatic stellate cells. Additionally, accumulated hydrophobic BAs induce mitochondrial ROS bursts and inhibit CPT1 α-mediated fatty acid oxidation (26). These cascades ultimately lead to hepatocellular steatosis, inflammation, and fibrosis, which constitute the core pathological mechanisms of aging-related MAFLD.

#### 4.2.3 Epigenetic modification

Host epigenetic programs dynamically interact with gut microflora through multi-level regulation. At the histone modification level, histone deacetylase 3 (HDAC3) maintains microbiota homeostasis through spatiotemporal regulation of intestinal epithelial antibacterial programs. Gut-specific HDAC3 knockout mice have decreased ileal α-defensin mRNA expression, resulting in increased proteobacteria abundance, while the microbiota metabolite butyric acid promotes Treg differentiation by inhibiting HDAC3 activity (112, 113). Silent mating type Information Regulation2 homolog 1(SIRT1) deficiency exacerbates fatty diet-induced hepatic lipid deposition (114, 115) by decreasing the ratio of secondary to primary BAs and inhibiting ileal FXR signaling. Chromatin remodeling mechanisms further drive the coevolution of the gut barrier and microbiota. Chromodomain Helicase DNA Binding protein 1(CHD1) deletion leads to a reduced expression of the TJ protein Claudin-3, and intestinal permeability is positively correlated with γ-proteobacteria abundance (116). Lysine-Specific Demethylase 5(KDM5/JARID1) regulates ZO-1 transcription through histone H3 lysine 4 trimethylation(H3K4me3) modification, and its intestinal epithelial-specific knockout increases LPS levels in the portal vein and activates the TLR4/MyD88 pathway in the liver (117). Non-coding RNA mediate precise regulation across species. Host miR-122-5p targets E. coli through exosomes, inhibits flagellin synthesis, and reduces bacterial biofilm formation capacity (118). Clinical cohorts have shown a significant negative correlation between fecal miR-21-5p levels and Enterobacteriaceae abundance (119) in patients with MAFLD. The intergenerational effects of environmental stress highlight the importance of epigenetic memories. Dexamethasone exposure during pregnancy induces Cytosine-phosphate-Guanine (CpG) island hypermethylation in the promoter region of the solute carrier family 5 member 8 (SLC5A8/SMCT1) gene in offspring mice, resulting in decreased SCFAs transporter expression, accompanied by an imbalance in the Bacteroides/Firmicutes ratio and an increased risk of liver steatosis in adulthood (120). These findings reveal the pivotal role of epigenetic regulation in the gut-liver axis, providing new targets for intervention in metabolic liver disease.

#### 4.2.4 Genetic polymorphisms and immune cells

Genome-wide association studies (GWAS) have revealed that carriers of the PNPLA3 rs738409 risk allele have an increased abundance of Prevotella in their colonic contents and an encoded alcohol dehydrogenase that catalyzes endogenous ethanol production and promotes lipid droplet deposition by inhibiting hepatocyte mitochondrial complex I activity (121, 122). Similarly, the TM6SF2 rs58542926 T allele results in a decreased abundance of the Bacteroides phylum, decreased synthesis of the secondary BA ursodeoxycholic acid (UDCA), impaired FXR-mediated carnitine palmitoyltransferase 1(CPT1) α expression, and activation of the NACHT, LRR, and PYD domains-containing protein 3 (NLRP3) inflammasome (123).

**Figure 3:**
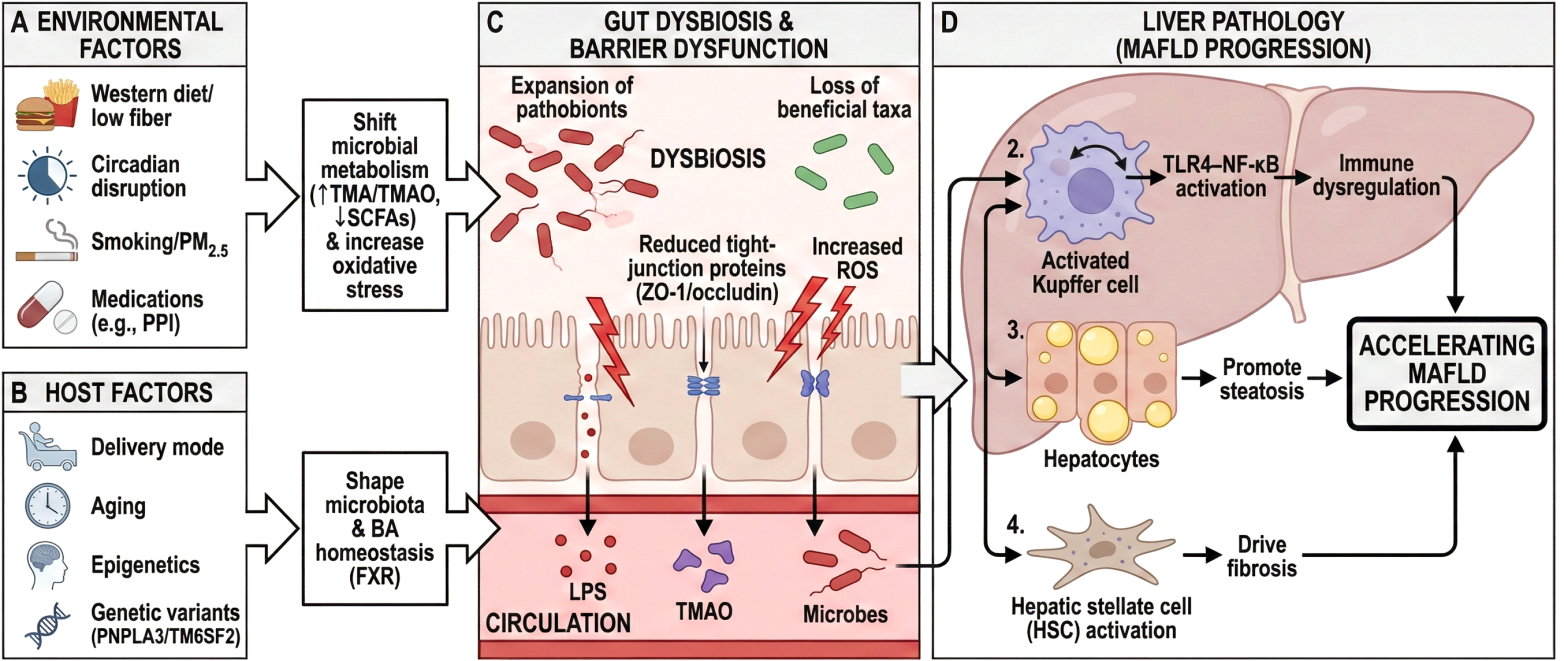
Drivers of gut dysbiosis–barrier dysfunction and their contribution to MAFLD proinflammatory flammatorytors (Western diet/low fiber, circadian disruption, smoking/PM2.5, and medications) shift microbial metabolism (↑TMA/TMAO, ↓SCFAs) and increase oxidative stress, predisposing patients to barrier leakage. **(B)** Host factors (delivery mode, aging, epigenetics, and genetic variants, such as PNPLA3/TM6SF2) further shape the microbiota and bile acid homeostasis. **(C)** Dysbiosis features expansion of pathobionts and loss of beneficial taxa, reduced tight-junction proteins (ZO-1/occludin), increased ROS, and translocation of LPS/TMAO and microbes into the circulation. **(D)** In the liver, these gut-derived signals activate Kupffer cells and inflammatory pathways (TLR4–NF-κB), promote steatosis and immune dysregulation, and drive fibrosis via hepatic stellate cell activation, thereby accelerating MAFLD progression. MAFLD, metabolic dysfunction–associated fatty liver disease; TMA/TMAO, trimethylamine/trimethylamine N-oxide; SCFAs, short-chain fatty acids; LPS, lipopolysaccharide; ROS, reactive oxygen species; BA, bile acids; FXR, farnesoid X receptor; PPI, proton pump inhibitor; HSC, hepatic stellate cell.

Gut-derived Tregs form immune homeostasis loops in response to microbiota metabolites. Butyric acid (≥ 50 μM) activates the G protein-coupled receptor 109A (GPR109A/HM74A) receptor on Tregs, induces C-C chemokine receptor type 9 (CCR9) expression, drives Treg migration to the lamina propria of the colon, and promotes IL-10 secretion (124–126) through histone H3 lysine 27 acetylation(H3K27ac) modification. This process inhibits hepatic CD8+ T cell proliferation and TNF-α release. Simultaneous mechanistic studies suggest that butyric acid upregulates forkhead box protein P3(Foxp3) expression by inhibiting HDAC3 activity in liver macrophages and blocks TLR4/NF-κB pathway overactivation (127) through suppressor of cytokine signaling 3(SOCS3)-mediated ubiquitination degradation. This regulatory axis is impaired in patients with MAFLD. Serum butyrate levels < 15 μM are associated with a decreased hepatic Treg/Teff ratio and are significantly negatively correlated with the lobular inflammation score and fibrosis stage (128, 129)(**Figure 3**).In summary, MAFLD-relevant dysbiosis is shaped by a convergence of exogenous exposures (dietary patterns, circadian disruption, smoking, air pollution, and medications) and endogenous host determinants (early life microbial seeding, aging, epigenetic programs, and genetic–immune context). Although these factors differ in timing and intensity, they tend to converge on common downstream consequences, including reduced beneficial metabolite output, impaired barrier integrity, and heightened portal inflammatory signaling. Conceptualizing risk factors through shared microbiota-barrier-liver nodes helps explain clinical heterogeneity and identifies modifiable entry points for prevention.

## 5 Molecular mechanism of MAFLD driven by the “enterobacteria-liver axis”

### 5.1 Gut mmicrobiomei

#### 5.1.1 LPS

Serum LPS levels are significantly elevated in patients with MAFLD, driving disease progression through a dual mechanism. First, LPS directly activates intrahepatic inflammation in the liver. It binds with high affinity to the toll-like receptor 4–myeloid differentiation factor 2 (TLR4-MD2) complex on the surface of hepatocytes and Kupffer cells, triggers myeloid differentiation primary response 88(MyD88) -dependent signaling pathways, induces IL-6 and IL-1β secretion, and directly leads to hepatocyte apoptosis and MASH deterioration (130). Serum co-receptor lipopolysaccharide-binding protein (LBP) levels are abnormally elevated in patients with MAFLD, and LBP gene knockout significantly improves liver lipid deposition and insulin resistance (131, 132). Additionally, LPS activates the NLRP3 inflammatory body through the TLR4/TRIF pathway, promotes caspase-1 self-cleavage and release of the gasdermin D N-terminal domain (GSDMD-N), drives mature IL-1β production, and intensifies the intrahepatic inflammatory cascade (133). Second, LPS indirectly aggravates liver injury by disrupting gut-liver axis homeostasis, activating the intestinal TLR4/MyD88 signaling axis, and upregulating myosin light chain kinase, resulting in decreased expression of the TJ protein occludin and significantly increasing intestinal permeability (134). Increased LPS levels in the portal vein aggravate the disease through a dual pathway: 1) activation of the NF-κB pathway in Kupffer cells, promotion of TGF-β1 secretion, induction of hepatic stellate cell activation, and collagen deposition (135); and 2) inhibition of Peroxisome Proliferator-Activated Receptor Alpha(PPARα) signaling in hepatocytes, reduction of fatty acid β-oxidation, and upregulation of sterol regulatory element-binding protein 1c(SREBP-1c143), a key factor in lipid generation. Eventually, a self-reinforcing vicious cycle develops between the gut and liver. Intrahepatic inflammatory factors (e.g., TNF-α) inhibit intestinal epithelial repair through portal feedback, resulting in a significant positive correlation between the abundance of LPS-producing proteobacteria in the gut and liver fibrosis stage (136). This cycle of “intestinal leakage-LPS into the liver-liver damage” is the core driving force for MAFLD progression to end-stage liver disease, providing a theoretical basis for precise treatment targeting the LPS/TLR4 pathway.

#### 5.1.2 Peptidoglycans

Peptidoglycans (PGN) possess immune recognition properties owing to their structural heterogeneity. The cell walls of gram-positive bacteria are approximately 50–100 nm thick, and the lipoteichoic acid in their cell walls is crosslinked with PGN to form a highly crosslinked three-dimensional network structure. In contrast, the cell walls of gram-negative bacteria (3–8 nm thin layer) are loosely anchored to the outer membrane (137, 138) by the Braun lipoprotein. This structural difference results in a higher PGN release efficiency in gram-positive bacteria than in gram-negative bacteria and significantly affects the activation threshold of the host pattern recognition receptor (139). In the HFD model, decreased fasting glucose and triglyceride (TG) deposition in TLR2^-/-mice were directly correlated with a decreased abundance of PGN-producing Lactobacillus (140). However, in the methionine- and choline-deficiency (MCD) model, isogenic defects resulted in increased liver inflammation scores, associated with increased PGN release and decreased levels of short-chain fatty acids (SCFAs) in Enterobacteriaceae, suggesting that dietary intervention dynamically modulates TLR2 signaling sensitivity (141) by reshaping the metabolic profile of the microbiota. PGN activates nucleotide-binding oligomerization domain-containing protein 1/2(NOD1/2) receptors with high affinity, synergistically triggering the NF-κB and MAPK pathways to drive a proinflammatory factor storm. NOD1/2 double-knockout mice have significantly decreased TG content and inflammatory cell infiltration area, confirming the key role of this pathway in lipid metabolism disorders (142, 143).

#### 5.1.3 Bacterial DNA

When microbial (bacterial) DNA is internalized by immune cells and trafficked to endosomal compartments, its unmethylated CpG motifs (5′-CpG-3′) can be recognized by toll-like receptor 9 (TLR9, an endosomal DNA-sensing PRR), initiating downstream signaling. The continued activation of extracellular signal-regulated kinase 1/2 (ERK1/2) and c-Jun N-terminal kinase (JNK) in the MAPK pathway has been reported in experimental systems to promote macrophage secretion of TNF-α and IL-12, while the NF-κB pathway can synergistically facilitate NLRP3 inflammasome assembly by accelerating inhibitor of kappa B alpha (IκBα) degradation and enhancing nuclear factor kappa B subunit p65 (p65) nuclear translocation, thereby providing mechanistic support for inflammation that may contribute to progression from MAFLD to MASH in animal models (144, 145). Notably, in preclinical studies, TLR9 inhibits interferon regulatory factor 7 (IRF7) phosphorylation via the IκB kinase α (IKKα)–microtubule-associated protein 1A/1B-light chain 3 (LC3) complex, reducing type I interferon synthesis and forming a “proinflammatoryanti-interferon” imbalance that may exacerbate metabolic inflammation by blunting antiviral responses (146).

In the methionine–choline deficient diet (MCD) feeding model, activation of the TLR9–MyD88 axis in Kupffer cells is associated with increased IL-1β secretion and has been linked to hepatocyte injury markers such as LDH release through caspase-1–dependent inflammatory cell death signaling in this model (147). Consistent with a functional role in mice, TLR9^-/- mice show reduced steatosis area, portal vein fibrosis score, and hepatic hydroxyproline content, supporting that TLR9–MyD88 signaling is an important contributor to MASH-like disease severity in this experimental setting, rather than establishing it as the sole driver in humans (148).

Bacterial DNA may also contribute to liver injury through broader immune reprogramming. Abnormal B cell activation promotes immunoglobulin G subclass 2c (IgG2c) secretion, leading to immune complex deposition and activation of the complement C3a–C5a axis. NK cells can induce hepatocyte apoptosis through perforin and Fas ligand/Fas receptor (Fas/FasL) signaling and, together with interferon-gamma (IFN-γ) secretion, may amplify inflammatory cascades (149, 150). In an early translational study, the TLR9 antagonist oligodeoxynucleotide (ODN) TTAGGG (5 mg/kg) reduced serum alanine aminotransferase (ALT) levels and improved NAS scores after four weeks of treatment; however, in patients, plasma bacterial DNA levels were reported to be strongly correlated with fibrosis stage, which supports clinical association but does not establish causality (151).

#### 5.1.4 Bacterial outer membrane vesicles

**Figure 4:**
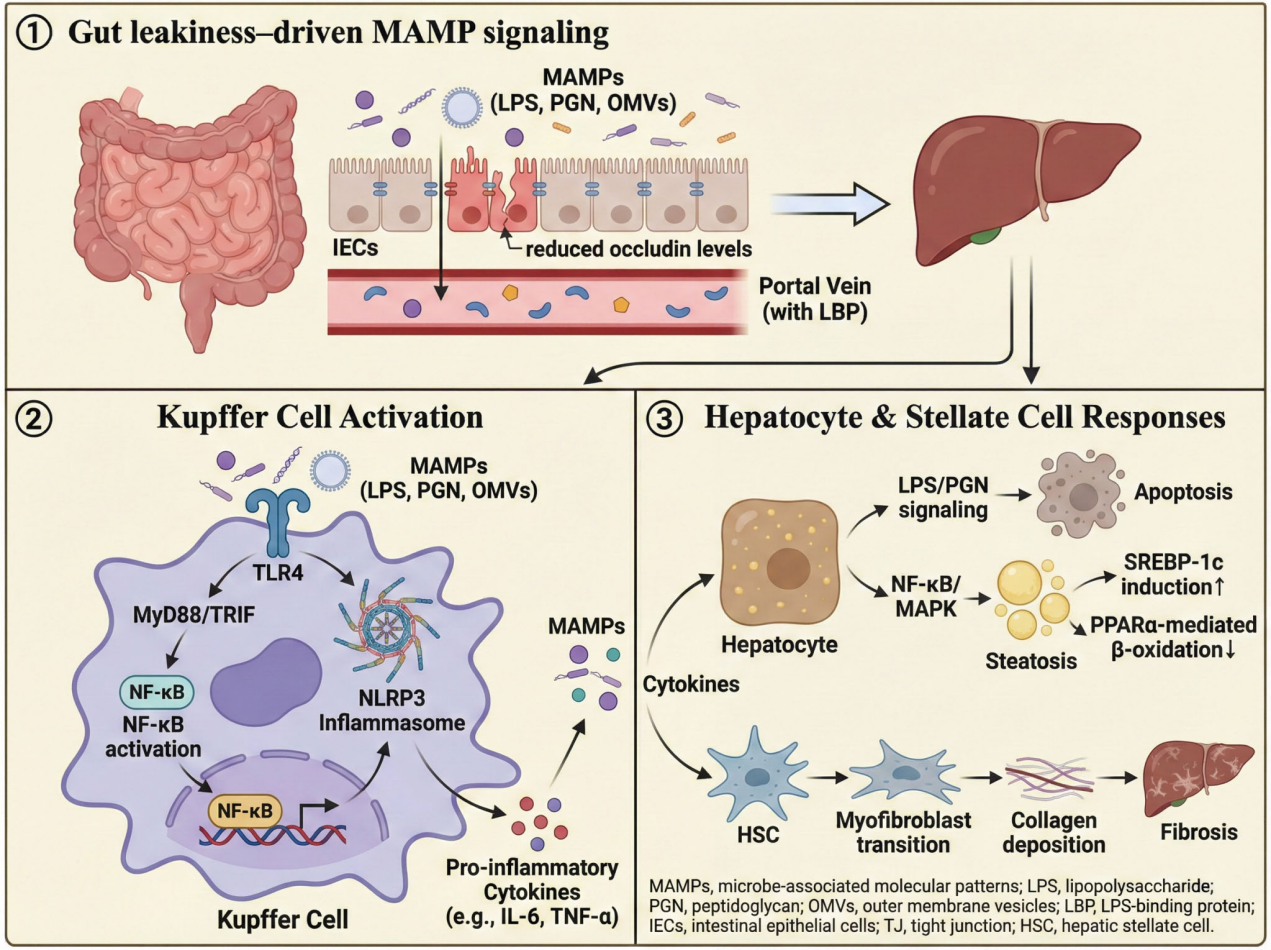
Gut leakiness–driven MAMP signaling promotes hepatic inflammation and fibrosis. Dysbiosis increases luminal microbial products (LPS, PGN, CpG-rich bacterial DNA, and OMVs) and disrupts epithelial tight junctions (e.g., reduced occludin levels), leading to increased intestinal permeability. These MAMPs enter the portal vein (often via LBP) and activate the hepatic innate immunity. In Kupffer cells, TLR4 signaling (MyD88/TRIF) drives NF-κB activation and inflammasome responses (NLRP3/caspase pathways), increasing the levels of pro-inflammatory cytokines. In hepatocytes, LPS/PGN signaling cooperates with NF-κB/MAPK to promote apoptosis and steatosis (e.g., such as SREBP-1c induction and reduced PPARα-mediated β-oxidation). Cytokine output activates hepatic stellate cells, resulting in myofibroblast transition and collagen deposition, thereby accelerating fibrosis. MAMPs, microbe-associated molecular patterns; LPS, lipopolysaccharide; PGN, peptidoglycan; OMVs, outer membrane vesicles; LBP, LPS-binding protein; IECs, intestinal epithelial cells; TJ, tight junction; HSC, hepatic stellaproinflammatory lammatoryta-derived metabolites and endotoxin drive the gut–liver axis in MAFLD. Dietary fiber, choline/L-carnitine, and ethanol shape dysbiotic microbiota, shifting SCFAs, TMA/TMAO, bile acids (BAs), lipopolysaccharides (LPS), and increased ethanol loss. The loss of tight junction (TJ) proteins (ZO-1/occludin) promotes leaky gut and portal delivery of microbial products. In the liver, TMAO–FXR/BA dysregulation and SCFA signaling (GPR43–AMPK/ACC; ACSS2) reprogram lipid metabolism, whereas ethanol (ADH/CYP2E1) generates ROS. LPS activates Kupffer cell TLR4–MyD88/TRIF–NF-κB to induce TNF-α, IL-6, and IL-1β, driving hepatic stellate cell (HSC) activation and fibrosis. The intervention nodes included OM-101, OCA, INT-777, TAK-242, and HDL3. SCFAs, short-chain fatty acids; TMA, trimethylamine; TMAO, trimethylamine N-oxide; BAs, bile acids; LPS, lipopolysaccharide; TJ, tight junction; ZO-1, zonula occludens-1; FMO3, flavin-containing monooxygenase 3; FXR, farnesoid X receptor; GPR43, G protein–coupled receptor 43 (FFAR2); AMPK, AMP-activated protein kinase; ACC, acetyl-CoA carboxylase; ACSS2, acyl-CoA synthetase short-chain family member 2; ADH, alcohol dehydrogenase; CYP2E1, cytochrome P450 2E1; ROS, reactive oxygen species; TLR4, Toll-like receptor 4; MyD88, myeloid differentiation primary response 88; TRIF, TIR-domain–containing adaptor-inducing interferon-β; NF-κB, nuclear factor kappa B; TNF-α, tumor necrosis factor alpha; IL-6, interleukin 6; IL-1β, interleukin 1 beta; HSC, hepatic stellate cell; OM-101, occludin mimetic; OCA, obeticholic acid; INT-777, TGR5 agonist; TAK-242, TLR4 inhibitor (resatorvid); HDL3, high-density lipoprotein 3.

Outer membrane vesicles (OMVs) are nanosized bilayer membrane vesicles with a diameter of 20–300 nm secreted by gram-negative (such as Escherichia coli) and some gram-positive bacteria. OMVs completely encapsulate LPS, virulent proteins, and circular DNA. OMVs deliver LPS directly to the cytoplasm of host cells through a membrane fusion-mediated Trojan horse mechanism, activating caspase-11-dependent non-classical inflammatory bodies and driving increased IL-1β secretion (152). In the gut microenvironment, OMVs are activated via a dual receptor coactivation pattern: MyD88-dependent signaling triggered by the TLR4-MD2 complex in intestinal epithelial cells and activation of NLRP3 inflammatory bodies in macrophages, resulting in the spatiotemporal co-release of TNF-α and IL-6 and an “inflammatory storm” (153, 154). This multi-target effect makes OMVs critical “inflammation amplifiers” for disrupting the enterohepatic axis in MAFLD. Clinical cohort studies have demonstrated a positive correlation between fecal OMV concentrations and serum ALT levels in patients with MAFLD, and liver tissue OMVs are highly localized within fibrotic regions (155, 156). At present, the molecular pathway by which OMVs affect host-microbiota interactions by regulating gut barrier TJ proteins and BA metabolism requires systematic analysis. Nano-intervention strategies based on OMV component editing may provide new ideas for remodeling the gut-liver immune homeostasis (**Figure 4**).

### 5.2 Metabolite-mediated pathological cascade

SCFAs, TMAO, and BAs produced by the metabolic reprogramming of intestinal microorganisms constitute the core medium of the gut-liver axis dialogue. SCFAs are transported to the liver via the portal vein, activate G protein-coupled receptor 43(GPR43) receptors, and inhibit histone deacetylase (HDAC) activity in hepatocytes, dually regulating PPARγ-mediated glycolipid homeostasis (157–159). TMAO promotes cytochrome P450 family 7 subfamily A member 1(CYP7A1)-dependent BA synthesis by activating the FXR/TGR5 signaling axis in hepatic stellate cells and induces NLRP3 inflammasome assembly (160). BAs inhibit sterol regulatory element-binding protein 1c(SREBP-1c) expression through the farnesoid X receptor (FXR)-small heterodimer partner (SHP) pathway and reduce TG deposition in the liver. Temporal and spatial activation of its receptor, pregnane X receptor (PXR), may coordinate gut barrier TJ protein expression with immune tolerance in liver Kupffer cells (161, 162). Clinical cohort studies have shown that when the total fecal SCFAs in patients with MAFLD decrease to 0, plasma TMAO levels are positively correlated with liver NAS scores, and serum DCA concentrations are negatively correlated with the fibrosis stage (163). Gene-editing strategies should target microbial metabolite synthases or provide precise intervention windows for remodeling the gut-liver metabolism-immune cross-talk.

#### 5.2.1 SCFAs

SCFAs (acetate, propionate, and butyrate) are the core metabolites of dietary fiber that are fermented by the gut microbiota. Approximately 95% of SCFAs enter the liver metabolic pool after absorption through the portal vein, 30–40% of which are converted into glycogen precursors, 10% are catalyzed by acyl-coA synthetase short-chain family member 2(ACSS2) to generate acetyl coenzyme A to participate in lipid neogenesis, and the remaining act on cells via G protein-coupled receptor 41/43(GPR41/43). Gut-brain-liver tripartite dialogue: the activation of intestinal L cells increases GLP-1 secretion and prolongs peptide YY(PYY) half-life, regulating food intake by inhibiting ghrelin (164). However, an abnormally high expression of GLP-1/PYY in the HFD model increases the risk of hepatic lipid accumulation via the activation of agouti-related protein/neuropeptide Y(AgRP/NPY) neurons in the hypothalamus (165). SCFAs exhibit concentration-dependent metabolic reprogramming in the liver. Physiological concentrations (50–200 μM) of propionate inhibit acetyl-CoA carboxylase (ACC) activity through AMP-activated protein kinase α subunit threonine 172(AMPKα Thr172) phosphorylation, decrease malonyl-CoA levels, and block fatty acid elongation. Supraphysiological concentrations (> 350 μM) of acetate drive the upregulation of fatty acid synthase (FASN) expression and promote lipid droplet formation (166, 167). Butyrate acts as an HDAC inhibitor and increases the acetylation of the adiponectin gene H3K27. Simultaneously, apparent silencing is achieved by methylation of the CpG island in the resistin promoter region, synergistically improving insulin sensitivity (168). Clinical cohorts have shown a decrease in total SCFAs in the portal vein of patients with MAFLD and a negative correlation between the butyrate ratio and the degree of hepatic steatosis, suggesting the need to develop strategies to precisely regulate SCFA composition/concentration based on individualized metabolic phenotypes (169).

#### 5.2.2 BAs

BAs convert cholesterol to primary BAs (CA/CDCA) via classical (CYP7A1) and alternative (CYP27A1/CYP7B1) pathways, which are secreted into the gut via glycine and taurine conjugation reactions. The gut microbiota converts primary BAs to secondary BAs (DCA/LCA) using BSH and 7α-dehydroxylase and maintains enterohepatic circulation 8–10 times a day via the apical sodium-dependent bile acid transporter (ASBT) (170). Patients with MAFLD have increased total serum BA concentrations, but the proportion of secondary BAs decreases significantly, with toxic taurine-bound CDCA accumulation (171). Secondary BAs drive pathological processes by abnormally regulating the FXR signaling axis. A reduced ability of hepatocyte CDCA to activate FXR leads to a failure of CYP7A1 negative feedback inhibition, triggering abnormal CA accumulation and inducing apoptosis through a mitochondrial cytochrome P450 family 2 subfamily E member 1(CYP2E1)-dependent ROS burst (172). INT-777 (10 μM), an activator of TGR5, inhibits the assembly of NLRP3 inflammatory bodies, decreases the polarization ratio of classically activated macrophages(M1)-type macrophages, and enhances the expression of bile salt export pump (BSEP), a key protein in biliary secretion, thus alleviating cholestatic injury (173, 174). Clinical cohorts showed a negative correlation between DCA/CA ratios and liver controlled attenuation parameter (CAP) values, while treatment with the FXR agonist obeticholic acid (OCA 25 mg/d) for 24 weeks reduced NAS scores, suggesting a new intervention strategy targeting the metabolic remodeling of BA or MAFLD (175).

#### 5.2.3 TMAO

TMAO is converted from dietary choline/L-carnitine to TMA by gut microbiota (such as Anaerococcus and Clostridium) through the CutC/D enzyme, which is oxidized by flavin-containing monooxygenase 3(FMO3) after entering the liver through the portal vein. Clinical studies have shown that patients with MAFLD have elevated serum TMAO levels compared to healthy individuals and a geometrically increased risk of advanced liver fibrosis (176). Animal experiments further confirmed that HFD-enriched CutC-positive microbiota increased intestinal choline-TMA conversion efficiency, decreased liver phosphatidylcholine bioavailability, aggravated very low-density lipoprotein (VLDL) secretion disorders, and increased hepatic lipid accumulation (177). However, the mechanism underlying the relationship between TMAO and the onset and development of MAFLD remains unclear. TMAO accelerated the degradation of insulin-induced gene 2(Insig-2) and increased the nuclear translocation efficiency of SREBP-1c through the ubiquitin-proteasome system. Concurrently, TMAO induced enhancer of zeste homolog 2(EZH2) to mediate H3K27me3 modification of the PPARα promoter region, inhibit CPT1A transcription, and lead to a decrease in the mitochondrial fatty acid oxidation rate (178). Further studies have shown that adipose tissue macrophages are the key targets of TMAO’s proinflammatory effect of TMAO. TMAO promotes the conversion of adipose tissue macrophages to the proinflammatory M1 phenotype through the STAT3 Tyr705 site and inhibits the expression of the alternatively activated macrophage (M2) marker arginase 1(Arg1), resulting in the formation of a chronic inflammatory microenvironment (179, 180). Furthermore, TMAO disrupts BA homeostasis by antagonizing FXR signaling. *In vitro* experiments have shown that 50 μM TMAO reduces FXR binding to the BSEP promoter of the BA efflux pump, resulting in cholestasis in the liver (181). The corresponding DCA further inhibits FXR signaling and promotes lipid regeneration by activating TGR5 receptors on the hepatocyte membrane, forming a self-reinforcing loop of “TMAO→FXR inhibition →DCA accumulation → lipid toxicity exacerbation” (182). In hepatic stellate cells, TMAO promotes IL-1β secretion by activating NLRP3 inflammatory bodies and indirectly stimulates collagen deposition in the liver. TMAO in hepatic sinusoidal endothelial cells downregulates the TJ protein Claudin-5 expression, increases vascular permeability, and promotes lipid particle transendothelial transport (183). This cell-type-specific regulation suggests that TMAO may drive both liver inflammation and abnormal lipid distribution in the liver. However, the specific molecular targets of this compound require further investigation.

#### 5.2.4 Other gut microbiota metabolites

Choline, an essential nutrient, is exogenously derived from the diet and endogenously synthesized by phosphatidylethanolamine N-methyltransferase (PEMT). It completes the lipidation of apolipoprotein B-100 by generating phosphatidylcholine (PC), which drives the assembly of VLDL particles (184). A choline-deficient diet reduced hepatic PC synthesis to approximately one-third of that in the control group, resulting in a decrease in the VLDL secretion rate and accumulation of TG in the liver. This model has been widely used for MASH mechanism analysis (185). In patients with MAFLD, the abundance of Bacteroides increases, CutC enzyme activity increases the conversion efficiency of dietary choline to TMA, and TMA concentration in the portal vein significantly increases. TMAO reduces endogenous choline synthesis by half in the healthy control group by inhibiting H3K4me3 modification of the PEMT promoter region, with a double effect of “exogenous entrapment and endogenous inhibition” (186). Clinical cohort studies have demonstrated that patients with MAFLD exhibit decreased free plasma choline levels, elevated serum TMAO concentrations, and abnormal metabolic axes that drive lipid neogenesis and inflammatory cascades (187) through the activation of SREBP-1c and NLRP3 inflammatory bodies. Glycerophosphocholine supplementation (2 g/d) for 12 weeks reduced liver fat content and improved NAS scores, suggesting that the precise regulation of choline metabolism is a potential therapeutic approach or a novel intervention strategy for MAFLD (188).

Endogenous ethanol is mainly produced by the gut microbiota (such as Escherichia coli and Klebsiella pneumoniae) through pentose phosphate metabolism. Clinical studies have shown that ethanol-producing bacteria are more abundant in patients with MASH than in healthy individuals, and the concentration of ethanol in peripheral blood is significantly positively correlated with the liver fat fraction. Single-cell transcriptome analysis further confirmed that alcohol dehydrogenase 1B(ADH1B) and aldehyde dehydrogenase 2(ALDH2), key enzymes of ethanol metabolism, were abnormally upregulated in MASH livers, suggesting a surge in the liver metabolic load (189, 190). In addition, the signaling pathway related to ethanol metabolism in the liver showed abnormal activation. From the perspective of liver metabolism, ethanol exposure can aggravate lipid deposition via dual mechanisms. Ethanol promotes lipid neogenesis by activating liver SREBP-1c and its downstream target genes, ACCα and FASN. Conversely, an increased NADH/NAD+ ratio inhibits PPARα transcription activity, resulting in a decreased mitochondrial β-oxidation rate (191, 192). Furthermore, CYP2E1 catalyzes the oxidation of ethanol to produce large amounts of acetaldehyde and ROS, directly damaging hepatocyte DNA. In addition, ROS promote the secretion of IL-1β and IL-18 by activating NLRP3 inflammatory bodies, initiating neutrophil infiltration and the activation of fibrosis precursor cells, and further aggravating liver inflammatory infiltration and fibrosis (86). At the gut microenvironment level, ethanol and its metabolites (especially acetaldehyde) can destroy intestinal epithelial TJ, resulting in impaired gut barrier function. For example, acetaldehyde (50 μM exposure for 24 hours) induced ubiquitination degradation of intestinal epithelial TJ proteins (occludin and claudin-1) and increased intestinal permeability.LPS enters the liver via the portal vein, activates the TLR4/MyD88 pathway in Kupffer cells, and upregulates TNF-α and IL-6 expression. These inflammatory factors can further aggravate cholestasis by inhibiting FXR signaling in hepatocytes and accelerating MAFLD progression.

Kupffer cells inhibit FXR signaling, leading to cholestasis (193). A clinical probiotic (Vivomixx^®^/Visbiome^®^ Strains Liquid [VSL #3]) intervention for 12 weeks reduced the abundance of ethanol-producing bacteria and the NAS score of the liver, proposing a novel direction for targeted microbiome-metabolic axis or MASH treatment (194, 195).

### 5.3 Microbial signal

#### 5.3.1 Impaired gut barrier: a prerequisite for alteration of the gut-liver axis

The gut barrier maintains host-microbiota interface homeostasis through TJ proteins, ZO-1 and occludin. Fecal zonulin concentrations are elevated in patients with MAFLD, suggesting early intestinal leakage (196). Animal models have demonstrated that gut barrier damage results in portal endotoxin and bacterial DNA translocation, thereby activating Kupffer cells. The TLR4/MyD88 pathway drives NF-κB nuclear translocation and NLRP3 inflammasome assembly, resulting in increased IL-1β secretion (197, 198).

Concentration-dependent bidirectional regulation of microbiota metabolites: secondary BAs (DCA, 10 μM) inhibit SREBP-1c expression by activating farnesoid X receptor (FXR). SCFAs enhance AMPK phosphorylation via G-protein coupled receptor 43(GPR43) at physiological concentrations (< 200 μM), but acetate promotes lipid neogenesis (199) by ACSS2-driven FASN expression at supraphysiological concentrations (> 350 μM). HDL3 neutralizes portal endotoxins via the apolipoprotein A-I binding domain and inhibits Kupffer cell activation in healthy livers. However, HDL3 depletion exacerbated hepatocyte apoptosis in an alcoholic liver injury model (200, 201). A clinical study showed that an oral occludin mimetic peptide (OM-101,50 mg/d) intervention for 8 weeks resulted in decreased fecal protein levels and improved NAS scores, whereas recombinant HDL3 infusion (80 mg/kg) significantly reduced serum LPS levels (202).

#### 5.3.2 Endotoxemia and TLR4

Gut barrier damage significantly increases the concentration of gram-negative bacterial endotoxin (LPS) in the portal vein, leading to metabolic endotoxemia (203). In experimental models, LPS triggers the MyD88-dependent signaling axis by binding to TLR4 (an innate receptor sensing bacterial LPS) on Kupffer cell surfaces, increases the nuclear translocation efficiency of NF-κB p65, promotes the release of TNF-α and IL-6, and is linked to increased serine 307 phosphorylation of insulin receptor substrate 1 (IRS-1) in hepatocytes, thereby contributing to insulin resistance in this setting (204–208).

A single-cell transcriptome study revealed that TLR4^+ macrophages expanded in the human MASH liver, secreted IL-6, and upregulated stearoyl-coA desaturase 1 (SCD1) expression through STAT3 Tyr705 phosphorylation, which is consistent with an association between this macrophage state and lipogenic signaling rather than establishing causality (209). The TRIF pathway downstream of TLR4 (a MyD88-independent adaptor route) coactivates the NLRP3 inflammasome, increasing caspase-1 activity and leading to GSDMD-mediated pyroptotic cell death signaling (210). A clinical cohort study revealed elevated serum soluble cluster of differentiation 14 (CD14) levels in patients with MASH, and a positive correlation was found between CD14 levels and liver fibrosis stage (211).From an “axis-to-cytokine” perspective, the gut–liver axis most directly reflects increased portal PAMP burden and consequent hepatic innate immune activation, which drives the local production of proinflammatory mediators such as TNF-α, IL-6, and IL-1β and couples hepatocellular injury to stellate-cell–mediated profibrotic responses. In contrast, the gut–spleen–liver axis highlights splenic immune amplification and the establishment of systemic cytokine tone, followed by immune cell trafficking and circulating inflammatory mediators that collectively shape the hepatic inflammatory microenvironment. This distinction helps reconcile locally triggered hepatic cytokine induction with broader, systemic immune dysregulation observed during disease progression.

**Figure 5:**
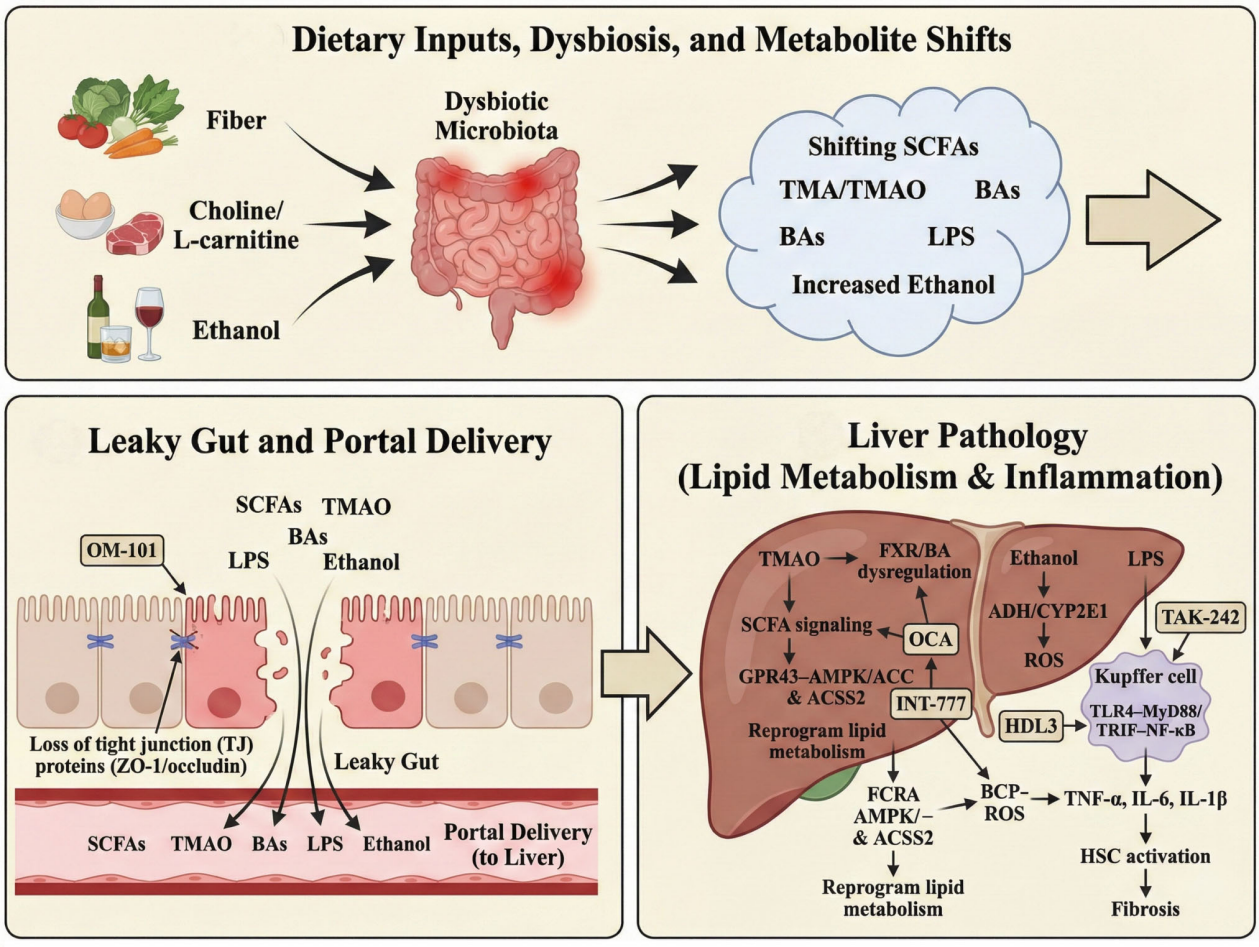
Schematic overview of the gut microbiota–liver axis in MAFLD. Dysbiosis alters microbial metabolites (e.g., SCFAs, bile acids, and TMA/TMAO) and promotes ethanol/acetaldehyde production and endotoxin (LPS) translocation across a leaky intestinal barrier into the portal vein. These signals modulate hepatocyte lipid metabolism and inflammatory pathways and activate Kupffer cells and hepatic stellate cells, ultimately driving fibrosis and collagen deposition. Representative intervention targets are highlighted (e.g., barrier protection, bile acid receptor signaling, and inhibition of LPS–TLR4–NF-κB/NLRP3 pathways).

A 6-week intervention with TAK-242 (1 mg/kg), a TLR4 inhibitor, in animal studies reduced portal LPS and improved NAS scores, supporting a causal contribution of gut-derived PAMPs–TLR4 signaling within the experimental model, while clinical causality in humans remains to be established (212, 213). Collectively, mechanistic evidence supports a model in which dysbiosis and barrier failure increase hepatic exposure to microbe-derived metabolites and molecular patterns, thereby reprogramming lipid handling and immune tone in the liver. Key effectors include bile acid signaling imbalance, SCFA depletion or context-dependent excess, and endotoxin-driven inflammatory cascades (e.g., TLR4–NF-κB/NLRP3), which together promote insulin resistance, steatosis, and fibrogenic progression. Framing these pathways as “metabolite–barrier–immune” modules provides a coherent bridge from microbial ecology to measurable hepatic phenotypes and therapeutic targets (**Figure 5**).

## 6 Interventional strategies targeting the “gut microbiota-liver axis”

**Figure 6:**
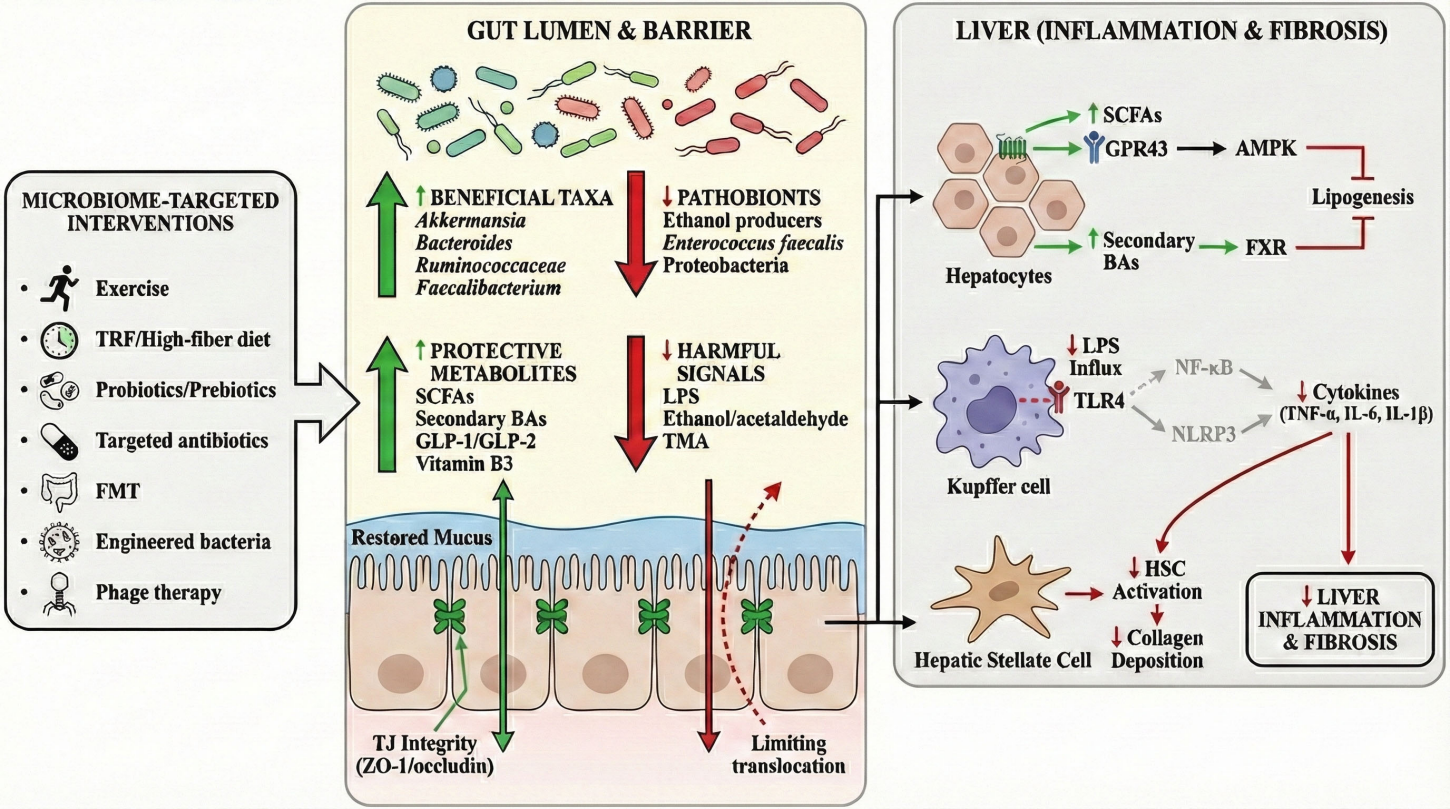
Microbiome-targeted interventions restore gut barrier function and attenuate liver inflammation and fibrosis through metabolite reprogramming. Lifestyle- and microbiota-directed interventions (exercise, TRF/high-fiber diet, probiotics/prebiotics, targeted antibiotics, FMT, engineered bacteria, and phage therapy) reshape gut communities, enriching beneficial taxa (e.g., *Akkermansia, Bacteroides*, Ruminococcaceae*, and Faecalibacterium*) and suppressing pathobionts (e.g., ethanol producers, *Enterococcus faecalis*, and Proteobacteria). This shift increases protective metabolites (SCFAs, secondary BAs, GLP-1/GLP-2, and vitamin B3) and reduces harmful signals (LPS, ethanol/acetaldehyde, and TMA), restoring mucus/TJ integrity (ZO-1/occludin) and limiting translocation. Downstream, hepatic SCFA–GPR43/AMPK and BA–FXR signaling restrain lipogenesis, while reduced LPS influx dampens Kupffer cell TLR4/NF-κB–NLRP3 activation and cytokines (TNF-α, IL-6, and IL-1β), lowering HSC activation, collagen deposition, and liver inflammation. TRF, time-restricted feeding; FMT, fecal microbiota transplantation; SCFAs, short-chain fatty acids; BAs, bile acids; DCA, deoxycholic acid; LCA, lithocholic acid; GLP, glucagon-like peptide; LPS, lipopolysaccharide; TMA, trimethylamine; TJ, tight junction; ZO-1, zonula occludens-1; GPR43, G protein–coupled receptor 43 (FFAR2); AMPK, AMP-activated protein kinase; FXR, farnesoid X receptor; TLR4, Toll-like receptor 4; NF-κB, nuclear factor kappa B; NLRP3, NLR family pyrin domain-containing 3; ACC, acetyl-CoA carboxylase; FASN, fatty acid synthase; TNF-α, tumor necrosis factor alpha; IL, interleukin; HSC, hepatic stellate cell.

Interventions targeting the gut–microbiota–liver axis can be organized into three actionable modules: (i) barrier restoration to reduce portal translocation of endotoxins and microbial products, (ii) metabolite rebalancing (e.g., SCFAs and bile acid signaling) to reset immunometabolic tone, and (iii) attenuation of innate immune overactivation triggered by gut-derived PAMPs. Lifestyle strategies (exercise, time-restricted feeding, and dietary composition) can be positioned as low-risk, scalable approaches that reshape microbial ecology and metabolite output (73–76). Ecological rebuilding (probiotics/prebiotics and FMT) may be considered when dysbiosis and barrier dysfunction dominate, whereas precision approaches (engineered bacteria and phage therapy) offer the potential to modulate specific taxa or functions. Importantly, intervention choice should match the dominant pathogenic bottleneck (barrier failure vs metabolite imbalance vs immune activation) and be evaluated using harmonized endpoints spanning permeability/portal endotoxin load, metabolite profiles, and hepatic inflammatory/fibrotic readouts (203–205, 209–211).(**Figure 6**).

### 6.1 Traditional gut microbiota interventions

#### 6.1.1 Exercise

Exercise improves MAFLD by regulating gut microbiota composition and function. Clinical data show that 12 weeks of moderate-intensity aerobic exercise (5 times a week, 60% VO_2_max) increases Akkermansia abundance, elevates serum butyrate concentrations, and reduces liver fat content in patients with MAFLD. Animal experiments confirmed that 5 weeks of aerobic resistance training (treadmill slope 8°, 60 min/day) increased the relative abundance of Bacteroidetes and inhibited the increase in the Firmicutes/Bacteroidetes ratio in HFD-fed rats. These changes were accompanied by a decrease in TG levels in the liver and an improvement in the insulin sensitivity index (214). Metagenomic analysis revealed that exercise upregulates the SCFA synthesis pathway in the microbiota and enhances the BA 7α-dehydroxylase gene cluster, promoting the production of secondary BAs (DCA) and activating hepatocyte FXR signaling (215). A randomized controlled trial (RCT) showed that 8 months of high-intensity interval training (HIIT) increased Ruminococcaceae abundance and decreased liver fibrosis scores in patients with MAFLD, and was significantly negatively correlated with fecal propionate concentration (216). Although further studies on the association between metabolic groups and host phenotypes of microbiota are needed, the available evidence supports the hypothesis that moderate-intensity exercise (e.g., brisk walking with an oxygen uptake of 12 mL/kg/min) for ≥ 150 min per week can maintain dominant colonization. Monitoring exercise intensity using wearable devices to optimize metabolic benefits is recommended (217, 218).

#### 6.1.2 Diet

Time-restricted feeding (TRF, 16:8 cycles) improves MAFLD by reconstructing spatiotemporal dynamics of the gut microbiota. Clinical data show that 8 weeks of TRF intervention increases Akkermansia muciniphila abundance in patients with MAFLD, significantly increases the relative abundance of Faecalibacterium prausnitzii, and restores microbiota α diversity (219). In an HFD model, TRF enhanced the amplitude of abundance fluctuations of Bacteroides during the active host phase, driving rhythmic butyrate synthesis and BA 7α-dehydroxylase activity (220).

Metagenomic identification of Ruminococcus sp. AM28–29LB is a core effector (abundance is negatively correlated with liver steatosis) that upregulates aldehyde oxidase 1(AOX1) expression in hepatocytes by synthesizing vitamin B3 and inhibiting nicotinamide (NAM)-dependent lipid accumulation (221). This bacterium also activates PPARγ in the intestinal epithelium by secreting acetic acid/butyric acid, enhances the expression of TJ ZO-1, and reduces intestinal permeability (29). ^A^ fecal bacterial transplantation (FMT) experiment confirmed that the TRF donor microbiota could reduce the total cholesterol in the livers of recipient sterile mice and reproduce the inhibitory effect of AOX1 expression, clarifying the causal relationship between the microbiota and host (67). In the future, synergistic interventions involving TRF, dietary fiber (such as inulin 10 g/d), and metabolic benefit optimization by targeting Ruminococcus functional gene cluster enhancement should be explored (222).

#### 6.1.3 Probiotics

Treating microbiota disorders presents multiple challenges. However, probiotic interventions have unique advantages. A multicenter RCT showed that a 12-week probiotic combination significantly reduced ALT and serum endotoxin (LPS) levels and increased Akkermansia muciniphila abundance (223). A liver biopsy confirmed that the proportion of hepatocyte ballooning in the probiotic group decreased significantly, and the NAS score improved significantly by ≥ 2 points (224). Mechanistic studies have revealed that Bifidobacterium longum BB536 activates AMPKα phosphorylation in hepatocytes by secreting acetic acid, inhibiting SREBP-1c-mediated lipid synthesis, and upregulating PPARα-driven fatty acid oxidation genes (225, 226). Animal experiments have shown strain-specific effects. BB536 decreased TG by approximately 40% in rats fed an HFD for 12 weeks, whereas Lactobacillus acidophilus NCFM decreased TG by only 20% (227). There is an urgent need to establish a strain-dose-treatment standardization protocol (recommended minimum effective dose 1 × 10^9^ CFU/day for ≥ 8 weeks) and analyze the interaction network of probiotics, bacteriophages, and dietary components using multi-component technology to promote personalized microecological treatment of MAFLD (228, 229).

#### 6.1.4 Prebiotics

Prebiotics (e.g., inulin and fructooligosaccharides) modify metabolic homeostasis by targeting the beneficial gut microbiota. A mouse model on an HFD showed that a 10% inulin intervention for 8 weeks significantly increased serum glucagon-like peptide-2 (GLP-2) concentrations and enhanced intestinal epithelial TJ protein (occludin) expression, resulting in a reduction in portal vein endotoxin (LPS^)^ (230). A metabolomic analysis confirmed that a fructooligosaccharide (5% w/w) intervention increased the proportion of the secondary BA decCA and inhibited SREBP-1c-mediated lipid synthesis (231) by activating the hepatic FXR signaling. Clinical studies have shown that 24 weeks of galactooligosaccharide (GOS, 5 g/d) intervention significantly reduced ALT levels, improved the homeostatic model assessment of insulin resistance(HOMA-IR) index, and markedly increased fecal butyrate concentrations (232) in patients with MAFLD. However, the available evidence is mostly based on surrogate endpoints, and there is an urgent need to verify the benefits histologically by performing liver biopsy and quantifying the liver fat fraction (233) using magnetic resonance imaging-proton density fat fraction (MRI-PDFF). In the future, metagenomics and spatial metabolomics technologies should be combined to analyze the spatiotemporal interactions between prebiotics and microbiota metabolites, and multicenter RCTs should be performed to verify the synergistic effects of GLP-1 receptor agonists.

#### 6.1.5 Antibiotics

Antibiotics, important tools for regulating gut microbiota, have a dose-dependent bidirectional effect in the treatment of MAFLD. Non-absorbable antibiotics, such as rifaximin, exert therapeutic effects through local regulation of microbial metabolism. In an HFD-induced MASH mouse model, rifaximin (50 mg/kg/d) decreased portal vein endotoxin levels, liver TG content, and collagen deposition area after 6 weeks of intervention. This mechanism involves FXR signal inhibition and secondary BA metabolic reprogramming (234–236). A RCT conducted by Gangarapu et al. showed that rifaximin (1200 mg/day for 4 weeks) reduced serum ALT levels and NAS scores in patients with MAFLD, which were associated with improved gut microbiota beta diversity index and CDCA/FXR pathway inhibition (237). However, Cobbold et al. found that a 400 mg bid for 6 weeks regimen increased the HOMA-IR index and decreased Bacteroides abundance, suggesting that long-term use of high doses may disrupt metabolic homeostasis (238). Triclosan (0.3 mg/kg/day) intervention specifically enriched Lactobacillus and Chaetospiraceae, increased the Bacteroides/Firmicutes ratio in MAFLD mice, and reduced the fatty degeneration area in the liver. Its efficacy is related to the upregulation of butyric acid synthesis gene expression, suggesting that triclosan can regulate the gut microbiota to treat MAFLD (239). However, antibiotics may have different effects, with mice treated with antibiotics exhibiting severe insulin resistance and an increased incidence and severity of histological lesions in MAFLD. These effects are associated with antibiotic-induced disturbances in the gut microbiota, resulting in changes in bifidobacteria and prevobacteria (240, 241). Therefore, antibiotics are a double-edged sword. On the one hand, antibiotics can regulate gut microbiota disorders by inactivating harmful bacteria to alleviate or treat the condition. However, long-term use of antibiotics may lead to bacterial drug resistance, affect the growth of beneficial bacteria in the body, and result in a new microbiota disorder.

#### 6.1.6 Fecal transplantation

FMT provides innovative intervention strategies for MAFLD treatment by transplanting functional microbial communities from healthy donor feces into the recipient’s intestines and reconstructing the microbiota ecology. Its mechanism involves many dimensions, such as microbiota remodeling, metabolite regulation, and signal transduction repair of the gut–liver axis. Evidence from animal models supports causality within the experimental setting. In animal experiments, FMT decreased the relative abundance of Bacteroides and increased the proportion of Firmicutes in an HFD-induced MAFLD mouse model. This microbiota remodeling was accompanied by improved gut barrier function, reduced portal endotoxin levels, decreased hepatic TNF-α concentrations, and a reduction in the hepatic steatosis area (242). While these findings support a causal contribution in rodents, translation to humans requires further clinical confirmation.

In a study of patients with metabolic syndrome, lean donor FMT increased peripheral insulin sensitivity and decreased hepatic TG levels in the recipients. Its efficacy is associated with an increased abundance of butyrate producers (243). Witjes et al. conducted a parallel-group study showing that the beta diversity of recipient microbiota approached that of the donor after 8 weeks of vegetarian donor FMT, with Bacteroides and Rothschella changing most distinctly (244). However, the clinical outcomes of MAFLD are modest and heterogeneous. Although the MAFLD activity scores did not differ statistically, histological analysis suggested a higher rate of improvement in lobular inflammation in the FMT group. Overall, the differences in MAFLD activity and fibrosis scores were not statistically significant, although the necroinflammation score (including lobular inflammation and hepatocellular ballooning) showed a trend toward improvement (245). Although previous clinical trials have demonstrated short-term improvements in intestinal permeability in patients with MAFLD, imaging assessments have shown that intrahepatic lipid content remained at baseline levels (246) during the 6-month follow-up period after FMT. Taken together, current human studies suggest associations and potential short-term physiological effects but are insufficient to establish causality or durable hepatic benefits without adequately powered randomized trials.

The current FMT technology has multiple potential risks. First, the lack of internationally unified standards for donor screening may lead to iatrogenic transmission of multidrug-resistant microorganisms (e.g., vancomycin-resistant enterococci) or latent pathogens (e.g., Clostridium difficile in the sporulation stage) (247). Second, drug-resistance genes carried by the microbiota can be integrated into the genome of host intestinal symbionts via conjugation transfer, and the β-lactamase gene expression level in the microbiota after intervention has been reported to be higher than that before transplantation (246). Third, metabolites of heterologous microorganisms may abnormally activate the Th17 pathway through pattern recognition receptors. In animal studies, IL-17A expression levels in the colon of recipient mice were increased compared those to in the control groups, and the inflammatory infiltration index in the portal vein area of the liver was also increased. Therefore, further studies are required to evaluate the long-term efficacy and safety of FMT in patients with MAFLD are required (246).

### 6.2 Synthetic biology and precision intervention

#### 6.2.1 Engineering bacteria design

Progress has been made in the design of engineered bacteria that target the core pathological link in the gut-liver axis. Animal model studies have revealed multiple mechanisms of engineering bacteria to treat MAFLD/metabolic dysfunction-associated steatotic liver disease (MAFLD). Recombinant Lactobacillus reuteri_ LR-01 increased intestinal mucosa thickness, increased ZO-1 expression, reduced portal vein endotoxin (LPS) levels, and significantly improved liver steatosis (248, 249) by activating the IL-22/STAT3 signaling axis. In addition, some engineered bacteria, by integrating butyrate synthase clusters (e.g., but operons), geometrically increased butyric acid concentrations in the colon, promoted fatty acid beta-oxidation through the PPARγ/AMPK dual pathway, and reduced the collagen deposition area (250) in a carbon tetrachloride-induced liver fibrosis model. Preclinical trials have confirmed the safety and metabolic regulatory potential of these engineered bacteria. Phase I trials showed that the orally-administered engineered bacteria Escherichia coli_ Nissle 1917 was well tolerated, had no abnormal levels of antibiotic resistance genes (ARGs), significantly increased the proportion of fecal secondary BAs (LCA), and decreased serum ALT levels (251, 252). Metabolic flux analysis revealed that acetyl-CoA pools in the liver of the engineered bacteria intervention group were enlarged, and the metabolic phenotypes of Kupffer cells were reshaped through succinate-mediated activation of HIF-1α (252, 253). Currently, there is an urgent need to address technical bottlenecks, such as the efficiency of colonization and genetic stability of engineered bacteria, and to develop metabolism-responsive intelligent strains using the clustered regularly interspaced short palindromic repeats-deactivated Cas9(CRISPR-dCas9) system, thereby promoting a new era of precision therapy targeting the gut-liver axis.

#### 6.2.2 Phage therapy

Complementing the metabolic regulation strategy of engineered bacteria, phage therapy blocks the vicious cycle of inflammatory signaling along the gut-liver axis by precisely eliminating the pathogenic bacteria. Although direct evidence for MAFLD is currently limited, alcoholic liver disease (ALD) research provides important references for elucidating its mechanisms. The cytolytic Enterococcus faecalis-specific phage cocktail designed by Duan et al. not only reduced the fatty degeneration area of liver tissue and serum ALT levels in ethanol-induced mouse models but also maintained the α diversity (254) of the gut microbiota. This finding is highly consistent with the mechanism of pathogen-specific proliferation, which induces metabolic disorders in MAFLD. Notably, clinical cohort analysis showed that for every 1 CFU/g increase in intestinal cytolytic E. faecalis abundance in patients with ALD, the 90-day mortality increased 2.3-fold, confirming its clinical urgency as a therapeutic target (254). Further mechanistic studies have shown that phage therapy ameliorates liver injury through a dual mechanism. First, the targeted elimination of TMA-producing proteobacteria (such as Klebsiella pneumoniae) reduces serum TMAO levels and inhibits the activation of the liver FMO3/NF-κB pathway. Second, reducing the secretion of cytolysin by cytolytic Enterococcus faecalis significantly increases intestinal epithelial TJ ZO-1 expression and reduces portal vein endotoxin (255). Despite its promising future, two major bottlenecks must be resolved before phage therapy can be applied in MAFLD. First, there are striking individual differences in the virulence genes carried by cytolytic Enterococcus faecalis (such as cylLL and esp) in the intestinal tract of patients with MAFLD, and a database of virulence genotype-phage sensitivity associations needs to be established. Second, whether the improvement in liver steatosis observed in the ALD model applies to the insulin resistance background specific to MAFLD (such as PI3K/Akt signaling suppression) remains to be verified. Two Phase I clinical trials (NCT05277350 and NCT0559984) are currently evaluating the effects of phage cocktails on gut microbiota homeostasis and liver elasticity in MAFLD patients. In summary, microbiota-targeted management of MAFLD includes lifestyle-based approaches (exercise and dietary timing/quality), ecological rebuilding strategies (probiotics/prebiotics and related modalities), and emerging precision tools that aim to modulate microbial functions and metabolite outputs. The most translatable pathway is to align interventions with the dominant pathogenic node in a given patient: restoring barrier competence, rebalancing bile acid/SCFA signaling, and dampening portal inflammatory inputs. Future studies should prioritize standardized endpoints and responder stratification to ensure that microbiota modulation translates into reproducible clinical benefits.

## 7 Challenges and prospects

The endotoxemia–TLR4 axis offers a pragmatic “mechanism-to-therapy” bridge by connecting a modifiable upstream driver (barrier-dependent portal LPS/PAMP exposure) (203). to druggable innate immune nodes (TLR4–MyD88/TRIF–NF-κB and inflammasome-linked effector programs) (204, 205, 209, 210). Looking forward, two translational priorities emerge: lowering portal endotoxin burden through barrier reinforcement and microbiota modulation and testing targeted inhibition of downstream innate pathways as proof-of-concept interventions (209, 210). In parallel, biomarkers such as soluble CD14 should be used for association-based stratification rather than causal claims (211).and durable benefit must be established in adequately powered randomized trials with standardized liver endpoints. The bi-directional regulation of the gut microenvironment and liver metabolic homeostasis has been gradually revealed with further analysis of the mechanism of the gut-microbiota-liver axis. Studies have shown that the gut microbiota plays a central regulatory role in the development of MAFLD by regulating key processes, such as energy metabolism, BA circulation, and gut-derived sex hormone secretion. Ectopic migration of microbiota-derived metabolites (e.g., LPS and TMA) caused by impairment of gut barrier function can trigger the “intestinal leak-endotoxemia-liver inflammation” cascade by activating the TLR4/MyD88-NLRP3 inflammatory body axis in the liver. Clinical evidence suggests that enterogenic genes can be detected in the portal blood of patients with acute liver injury.

The detection of 16S rDNA fragments of Enterobacteriaceae in the liver suggests a direct migration path between the gut microbiota and liver. However, the molecular mechanism of cross-tissue colonization requires further clarification (256).

The current study faced a dual-dimensional challenge. First, at the mechanistic level, gut microbiota metabolites exert complex concentration-dependent effects. For example, butyric acid inhibits lipid synthesis through AMPKα phosphorylation at < 200 μM, but drives fat deposition through ACSS2-mediated histone acetylation above 350 μM, the signal transition threshold of which has not been precisely defined (257, 258). At the technical level, it is difficult to simulate portal vein SCFA gradients using existing organoid models, and the single-cell transcriptome temporal resolution (6 h) cannot capture the 20-minute transient activation (259) of the TLR4/NF-κB pathway. Breakthrough technologies offer new tools for elucidating these mechanisms. CRISPR-labeled fluorescent reporter strains (e.g., mCherry marker_Bacteroides thetaiotaomicron_) combined with *in vivo* two-photon imaging allow for the first dynamic visualization of gut microbiota-liver migration and reveal that strain-specific fragments activate NLRP3 inflammasomes (260, 261) through c-type lectin domain family 4 member F(CLEC4F) receptor-mediated burial in Kupffer cells. Owing to the subtype heterogeneity of patients with MAFLD, constructing a comprehensive microbiome predictive model that integrates both viral and fungal groups to accurately identify the Bacteroides non-responder population is necessary (6). Future breakthroughs may include the development of a concentration-gradient chip combined with single-cell metabolic flux technologies to dissect the biphasic switching mechanisms of microbial metabolites, the design of CRISPR–dCas13-mediated suicide switch systems to ensure the genetic stability of engineered Escherichia coli Nissle 1917, and the establishment of a drug–bacterial interaction knowledge map to quantitatively predict adverse effects of PPAR-targeted therapy (e.g., rosiglitazone, 4 mg/day) (262). Through the integration of multi-omics datasets and AI-driven modeling, accurate mapping of “microbiota metabolism–liver disease phenotype–treatment response” may be achievable, thereby advancing hierarchical diagnosis and personalized therapy for MAFLD. Overall, the gut microbiota–liver axis provides an integrative framework linking environmental exposures and host susceptibility to hepatic metabolic inflammation through barrier integrity, microbial metabolites, and innate immune activation (263, 264), with additional layers of host–microbe signaling potentially mediated by glycoconjugates and related molecular interactions (265).

However, current evidence linking the gut–microbiota–liver axis to MAFLD/MASH is constrained by several limitations. First, many human studies remain observational and cross-sectional, with heterogeneous diagnostic criteria and insufficient control for diet and medications, which restricts causal inference; notably, dietary exposures such as coffee intake may independently associate with liver-related outcomes (266). Second, animal models (e.g., HFD- and MCD-based models) capture selected facets of disease and differ from human MAFLD in immune and metabolic contexts; thus, mechanistic causality demonstrated in rodents may not directly translate to humans. Third, microbiota-targeted interventions (probiotics, prebiotics, FMT, and engineered bacteria) vary substantially in donor/strain composition, dosing, duration, and endpoints, and clinical trials are often underpowered with short follow-up, limiting conclusions regarding durable hepatic outcomes; compositional features such as Erysipelotrichaceae may represent context-dependent contributors to host metabolic and inflammatory phenotypes (267), and regulation of intestinal microbiota has also been proposed as a key mechanism of natural medicines in metabolic disorders (268). Finally, multi-omics associations are vulnerable to batch effects and confounding, underscoring the need for standardized pipelines, pre-registered endpoints, and replication across independent cohorts and geographies. In parallel, fibrosis-related remodeling and its downstream consequences (e.g., ECM synthesis and progression toward advanced liver disease) require more rigorous longitudinal validation in humans (269).

## References

[1] Guo Z Wu D Mao R Yao Z Wu Q Lv W . Global burden of mafld, mafld related cirrhosis and mash related liver cancer from 1990 to 2021. Sci Rep. (2025) 15:7083. doi: 10.1038/s41598-025-91312-5, PMID: 40016310 PMC11868648

[2] Le MH Yeo YH Zou B Barnet S Henry L Cheung R . Forecasted 2040 global prevalence of nonalcoholic fatty liver disease using hierarchical bayesian approach. Clin Mol Hepatol. (2022) 28:841–50. doi: 10.3350/cmh.2022.0239, PMID: 36117442 PMC9597215

[3] Cao C Liu W Guo X Weng S Chen Y Luo Y . Identification and validation of efferocytosis-related biomarkers for the diagnosis of metabolic dysfunction-associated steatohepatitis based on bioinformatics analysis and machine learning. Front Immunol. (2024) 15:1460431. doi: 10.3389/fimmu.2024.1460431, PMID: 39497821 PMC11532026

[4] Bansal SK Bansal MB . Pathogenesis of masld and mash - role of insulin resistance and lipotoxicity. Aliment Pharmacol Ther. (2024) 59 Suppl 1:S10–22. doi: 10.1111/apt.17930, PMID: 38451123

[5] Vallianou NG Kounatidis D Psallida S Vythoulkas-Biotis N Adamou A Zachariadou T . Nafld/masld and the gut-liver axis: from pathogenesis to treatment options. Metabolites. (2024) 14:366. doi: 10.3390/metabo14070366, PMID: 39057689 PMC11278747

[6] Alghamdi W Mosli M Alqahtani SA . Gut microbiota in mafld: therapeutic and diagnostic implications. Ther Adv Endocrinol Metab. (2024) 15:1839355079. doi: 10.1177/20420188241242937, PMID: 38628492 PMC11020731

[7] Jasirwan C Muradi A Hasan I Simadibrata M Rinaldi I . Correlation of gut firmicutes/bacteroidetes ratio with fibrosis and steatosis stratified by body mass index in patients with non-alcoholic fatty liver disease. Biosci Microbiota Food Health. (2021) 40:50–8. doi: 10.12938/bmfh.2020-046, PMID: 33520569 PMC7817510

[8] Chen J Xiao Y Li D Zhang S Wu Y Zhang Q . New insights into the mechanisms of high-fat diet mediated gut microbiota in chronic diseases. Imeta. (2023) 2:e69. doi: 10.1002/imt2.69, PMID: 38868334 PMC10989969

[9] Datta S Pasham S Inavolu S Boini KM Koka S . Role of gut microbial metabolites in cardiovascular diseases-current insights and the road ahead. Int J Mol Sci. (2024) 25:10208. doi: 10.3390/ijms251810208, PMID: 39337693 PMC11432476

[10] Tan X Liu Y Long J Chen S Liao G Wu S . Trimethylamine n-oxide aggravates liver steatosis through modulation of bile acid metabolism and inhibition of farnesoid x receptor signaling in nonalcoholic fatty liver disease. Mol Nutr Food Res. (2019) 63:e1900257. doi: 10.1002/mnfr.201900257, PMID: 31095863

[11] Boroviak T Loos R Lombard P Okahara J Behr R Sasaki E . Lineage-specific profiling delineates the emergence and progression of naive pluripotency in mammalian embryogenesis. Dev Cell. (2015) 35:366–82. doi: 10.1016/j.devcel.2015.10.011, PMID: 26555056 PMC4643313

[12] Harkins JM Ahmad B . Anatomy, abdomen and pelvis, portal venous system (hepatic portal system). In: StatPearls [Internet]. Treasure Island (FL): StatPearls Publishing. (2025)., PMID: 32119476

[13] Compare D Coccoli P Rocco A Nardone OM De Maria S Carteni M . Gut--liver axis: the impact of gut microbiota on non alcoholic fatty liver disease. Nutr Metab Cardiovasc Dis. (2012) 22:471–6. doi: 10.1016/j.numecd.2012.02.007, PMID: 22546554

[14] Guo X Okpara ES Hu W Yan C Wang Y Liang Q . Interactive relationships between intestinal flora and bile acids. Int J Mol Sci. (2022) 23:8343. doi: 10.3390/ijms23158343, PMID: 35955473 PMC9368770

[15] Li X He M Yi X Lu X Zhu M Xue M . Short-chain fatty acids in nonalcoholic fatty liver disease: new prospects for short-chain fatty acids as therapeutic targets. Heliyon. (2024) 10:e26991. doi: 10.1016/j.heliyon.2024.e26991, PMID: 38486722 PMC10937592

[16] Seki E Park E Fujimoto J . Toll-like receptor signaling in liver regeneration, fibrosis and carcinogenesis. Hepatol Res. (2011) 41:597–610. doi: 10.1111/j.1872-034X.2011.00822.x, PMID: 21696522 PMC3754784

[17] Salim SY Soderholm JD . Importance of disrupted intestinal barrier in inflammatory bowel diseases. Inflammation Bowel Dis. (2011) 17:362–81. doi: 10.1002/ibd.21403, PMID: 20725949

[18] Turner JR . Intestinal mucosal barrier function in health and disease. Nat Rev Immunol. (2009) 9:799–809. doi: 10.1038/nri2653, PMID: 19855405

[19] Kurashima Y Kiyono H . Mucosal ecological network of epithelium and immune cells for gut homeostasis and tissue healing. Annu Rev Immunol. (2017) 35:119–47. doi: 10.1146/annurev-immunol-051116-052424, PMID: 28125357

[20] Suzuki T . Regulation of intestinal epithelial permeability by tight junctions. Cell Mol Life Sci. (2013) 70:631–59. doi: 10.1007/s00018-012-1070-x, PMID: 22782113 PMC11113843

[21] Furuse M Fujita K Hiiragi T Fujimoto K Tsukita S . Claudin-1 and -2: novel integral membrane proteins localizing at tight junctions with no sequence similarity to occludin. J Cell Biol. (1998) 141:1539–50. doi: 10.1083/jcb.141.7.1539, PMID: 9647647 PMC2132999

[22] Furuse M Hirase T Itoh M Nagafuchi A Yonemura S Tsukita S . Occludin: a novel integral membrane protein localizing at tight junctions. J Cell Biol. (1993) 123(6):1777–88. doi: 10.1083/jcb.123.6.1777, PMID: 8276896 PMC2290891

[23] Cereijido M Contreras RG Flores-Benitez D Flores-Maldonado C Larre I Ruiz A . New diseases derived or associated with the tight junction. Arch Med Res. (2007) 38:465–78. doi: 10.1016/j.arcmed.2007.02.003, PMID: 17560451

[24] Bauer H Zweimueller-Mayer J Steinbacher P Lametschwandtner A Bauer HC . The dual role of zonula occludens (zo) proteins. J BioMed Biotechnol. (2010) 2010:402593. doi: 10.1155/2010/402593, PMID: 20224657 PMC2836178

[25] Lee B Moon KM Kim CY . Tight junction in the intestinal epithelium: its association with diseases and regulation by phytochemicals. J Immunol Res. (2018) 2018:2645465. doi: 10.1155/2018/2645465, PMID: 30648119 PMC6311762

[26] Ghosh SS Wang J Yannie PJ Ghosh S . Intestinal barrier dysfunction, lps translocation, and disease development. J Endocr Soc. (2020) 4:bvz39. doi: 10.1210/jendso/bvz039, PMID: 32099951 PMC7033038

[27] Mohammad S Thiemermann C . Role of metabolic endotoxemia in systemic inflammation and potential interventions. Front Immunol. (2020) 11:594150. doi: 10.3389/fimmu.2020.594150, PMID: 33505393 PMC7829348

[28] Rinninella E Raoul P Cintoni M Franceschi F Miggiano G Gasbarrini A . What is the healthy gut microbiota composition? A changing ecosystem across age, environment, diet, and diseases. Microorganisms. (2019) 7:14. doi: 10.3390/microorganisms7010014, PMID: 30634578 PMC6351938

[29] Yan H Ajuwon KM . Butyrate modifies intestinal barrier function in ipec-j2 cells through a selective upregulation of tight junction proteins and activation of the akt signaling pathway. PloS One. (2017) 12:e179586. doi: 10.1371/journal.pone.0179586, PMID: 28654658 PMC5487041

[30] D’Hennezel E Abubucker S Murphy LO Cullen TW . Total lipopolysaccharide from the human gut microbiome silences toll-like receptor signaling. mSystems. (2017) 2:e00046–17. doi: 10.1128/mSystems.00046-17, PMID: 29152585 PMC5686520

[31] Ribatti D . Gut microbiota, intestinal permeability, and systemic inflammation: a narrative review. Comment. Intern Emerg Med. (2024) 19:1515–6. doi: 10.1007/s11739-024-03600-z, PMID: 38652233

[32] Mantis NJ Rol N Corthesy B . Secretory iga’s complex roles in immunity and mucosal homeostasis in the gut. Mucosal Immunol. (2011) 4:603–11. doi: 10.1038/mi.2011.41, PMID: 21975936 PMC3774538

[33] Cai J Sun L Gonzalez FJ . Gut microbiota-derived bile acids in intestinal immunity, inflammation, and tumorigenesis. Cell Host Microbe. (2022) 30:289–300. doi: 10.1016/j.chom.2022.02.004, PMID: 35271802 PMC8923532

[34] Dossa AY Escobar O Golden J Frey MR Ford HR Gayer CP . Bile acids regulate intestinal cell proliferation by modulating egfr and fxr signaling. Am J Physiol Gastrointest Liver Physiol. (2016) 310:G81–92. doi: 10.1152/ajpgi.00065.2015, PMID: 26608185 PMC4719061

[35] An J Liu Y Wang Y Fan R Hu X Zhang F . The role of intestinal mucosal barrier in autoimmune disease: a potential target. Front Immunol. (2022) 13:871713. doi: 10.3389/fimmu.2022.871713, PMID: 35844539 PMC9284064

[36] Naveed M Wang Y Yin X Chan M Aslam S Wang F . Purification, characterization and bactericidal action of lysozyme, isolated from bacillus subtillis bsn314: a disintegrating effect of lysozyme on gram-positive and gram-negative bacteria. Molecules. (2023) 28:1058. doi: 10.3390/molecules28031058, PMID: 36770725 PMC9919333

[37] Baars A Oosting A Knol J Garssen J van Bergenhenegouwen J . The gut microbiota as a therapeutic target in ibd and metabolic disease: a role for the bile acid receptors fxr and tgr5. Microorganisms. (2015) 3:641–66. doi: 10.3390/microorganisms3040641, PMID: 27682110 PMC5023267

[38] Mallesh S Ten HA Schneider R Schneiker B Efferz P Kalff JC . Sympathetic innervation modulates mucosal immune homeostasis and epithelial host defense. Cells. (2022) 11:2606. doi: 10.3390/cells11162606, PMID: 36010681 PMC9406312

[39] Koboziev I Karlsson F Grisham MB . Gut-associated lymphoid tissue, t cell trafficking, and chronic intestinal inflammation. Ann N Y Acad Sci. (2010) 1207 Suppl 1:E86–93. doi: 10.1111/j.1749-6632.2010.05711x, PMID: 20961311 PMC3075575

[40] Mabbott NA Donaldson DS Ohno H Williams IR Mahajan A . Microfold (m) cells: important immunosurveillance posts in the intestinal epithelium. Mucosal Immunol. (2013) 6:666–77. doi: 10.1038/mi.2013.30, PMID: 23695511 PMC3686595

[41] Bowman EP Kuklin NA Youngman KR Lazarus NH Kunkel EJ Pan J . The intestinal chemokine thymus-expressed chemokine (ccl25) attracts igaantibody-secreting cells. J Exp Med. (2002) 195:269–75. doi: 10.1084/jem.20010670, PMID: 11805153 PMC2193602

[42] van Dalen R Elsherbini A Harms M Alber S Stemmler R Peschel A . Secretory iga impacts the microbiota density in the human nose. Microbiome. (2023) 11:233. doi: 10.1186/s40168-023-01675-y, PMID: 37865781 PMC10589987

[43] Luo H Guo P Zhou Q . Role of tlr4/nf-kappab in damage to intestinal mucosa barrier function and bacterial translocation in rats exposed to hypoxia. PloS One. (2012) 7:e46291. doi: 10.1371/journal.pone.0046291, PMID: 23082119 PMC3474812

[44] Teunis C Nieuwdorp M Hanssen N . Interactions between tryptophan metabolism, the gut microbiome and the immune system as potential drivers of non-alcoholic fatty liver disease (nafld) and metabolic diseases. Metabolites. (2022) 12(6):514. doi: 10.3390/metabo12060514, PMID: 35736447 PMC9227929

[45] Castro-Dopico T Clatworthy MR . Igg and fcgamma receptors in intestinal immunity and inflammation. Front Immunol. (2019) 10:805. doi: 10.3389/fimmu.2019.00805, PMID: 31031776 PMC6473071

[46] Abenavoli L Scarlata G Scarpellini E Boccuto L Spagnuolo R Tilocca B . Metabolic-dysfunction-associated fatty liver disease and gut microbiota: from fatty liver to dysmetabolic syndrome. Med (Kaunas). (2023) 59:594. doi: 10.3390/medicina59030594, PMID: 36984595 PMC10054528

[47] Abenavoli L Gambardella ML Scarlata G Lenci I Baiocchi L Luzza F . The many faces of metabolic dysfunction-associated fatty liver disease treatment: from the mediterranean diet to fecal microbiota transplantation. Med (Kaunas). (2024) 60:563. doi: 10.3390/medicina60040563, PMID: 38674209 PMC11051743

[48] Ji J Sun J Li J Xie J Xi B Zhao M . Altered gut microbiome associated with metabolic-associated fatty liver disease in chinese children. Clin Nutr. (2024) 43:187–96. doi: 10.1016/j.clnu.2023.11.001, PMID: 38070210

[49] Rizzatti G Lopetuso LR Gibiino G Binda C Gasbarrini A . Proteobacteria: a common factor in human diseases. BioMed Res Int. (2017) 2017:9351507. doi: 10.1155/2017/9351507, PMID: 29230419 PMC5688358

[50] Zhang D Wang Q Li D Chen S Chen J Zhu X . Gut microbiome composition and metabolic activity in metabolic-associated fatty liver disease. Virulence. (2025) 16:2482158. doi: 10.1080/21505594.2025.2482158, PMID: 40122128 PMC11959907

[51] Zhou J Tripathi M Sinha RA Singh BK Yen PM . Gut microbiota and their metabolites in the progression of non-alcoholic fatty liver disease. Hepatoma Res. (2021) 7:11. doi: 10.20517/2394-5079.2020.134, PMID: 33490737 PMC7116620

[52] Schnabl B Damman CJ Carr RM . Metabolic dysfunction-associated steatotic liver disease and the gut microbiome: pathogenic insights and therapeutic innovations. J Clin Invest. (2025) 135:e186423. doi: 10.1172/JCI186423, PMID: 40166938 PMC11957707

[53] Christopherson MR Dawson JA Stevenson DM Cunningham AC Bramhacharya S Weimer PJ . Unique aspects of fiber degradation by the ruminal ethanologen ruminococcus albus 7 revealed by physiological and transcriptomic analysis. BMC Genomics. (2014) 15:1066. doi: 10.1186/1471-2164-15-1066, PMID: 25477200 PMC4300822

[54] Xu QY Ren TY Zhou YC Xu J Du LD Hong DY . Prevotella copri-produced 5-aminopentanoic acid promotes pediatric metabolic dysfunction-associated steatotic liver disease. Hepatobiliary Pancreat Dis Int. (2025) 24:303–15. doi: 10.1016/j.hbpd.2025.02.004, PMID: 40057459

[55] Bellanti F Lo BA Vendemiale G . Hepatic mitochondria-gut microbiota interactions in metabolism-associated fatty liver disease. Metabolites. (2023) 13:322. doi: 10.3390/metabo13030322, PMID: 36984762 PMC10057853

[56] Guo GJ Yao F Lu WP Xu HM . Gut microbiome and metabolic-associated fatty liver disease: current status and potential applications. World J Hepatol. (2023) 15:867–82. doi: 10.4254/wjh.v15.i7.867, PMID: 37547030 PMC10401411

[57] Chelakkot C Ghim J Ryu SH . Mechanisms regulating intestinal barrier integrity and its pathological implications. Exp Mol Med. (2018) 50:1–9. doi: 10.1038/s12276-018-0126-x, PMID: 30115904 PMC6095905

[58] Dubuquoy L Rousseaux C Thuru X Peyrin-Biroulet L Romano O Chavatte P . Ppargamma as a new therapeutic target in inflammatory bowel diseases. Gut. (2006) 55:1341–9. doi: 10.1136/gut.2006.093484, PMID: 16905700 PMC1860011

[59] Zhao J Zhao R Cheng L Yang J Zhu L . Peroxisome proliferator-activated receptor gamma activation promotes intestinal barrier function by improving mucus and tight junctions in a mouse colitis model. Dig Liver Dis. (2018) 50:1195–204. doi: 10.1016/j.dld.2018.04.016, PMID: 29891333

[60] Gavzy SJ Kensiski A Lee ZL Mongodin EF Ma B Bromberg JS . Bifidobacterium mechanisms of immune modulation and tolerance. Gut Microbes. (2023) 15:2291164. doi: 10.1080/19490976.2023.2291164, PMID: 38055306 PMC10730214

[61] De Filippo C Cavalieri D Di Paola M Ramazzotti M Poullet JB Massart S . Impact of diet in shaping gut microbiota revealed by a comparative study in children from europe and rural africa. Proc Natl Acad Sci U S A. (2010) 107:14691–6. doi: 10.1073/pnas.1005963107, PMID: 20679230 PMC2930426

[62] Christensen L Vuholm S Roager HM Nielsen DS Krych L Kristensen M . Prevotella abundance predicts weight loss success in healthy, overweight adults consuming a whole-grain diet ad libitum: a *post hoc* analysis of a 6-wk randomized controlled trial. J Nutr. (2019) 149:2174–81. doi: 10.1093/jn/nxz198, PMID: 31504699

[63] Clemente-Suarez VJ Beltran-Velasco AI Redondo-Florez L Martin-Rodriguez A Tornero-Aguilera JF . Global impacts of western diet and its effects on metabolism and health: a narrative review. Nutrients. (2023) 15:2749. doi: 10.3390/nu15122749, PMID: 37375654 PMC10302286

[64] Chiang J Ferrell JM . Bile acid receptors fxr and tgr5 signaling in fatty liver diseases and therapy. Am J Physiol Gastrointest Liver Physiol. (2020) 318:G554–73. doi: 10.1152/ajpgi.00223.2019, PMID: 31984784 PMC7099488

[65] Song KH Li T Owsley E Strom S Chiang JY . Bile acids activate fibroblast growth factor 19 signaling in human hepatocytes to inhibit cholesterol 7alpha-hydroxylase gene expression. Hepatology. (2009) 49:297–305. doi: 10.1002/hep.22627, PMID: 19085950 PMC2614454

[66] Sun P Wang M Liu YX Li L Chai X Zheng W . High-fat diet-disturbed gut microbiota-colonocyte interactions contribute to dysregulating peripheral tryptophan-kynurenine metabolism. Microbiome. (2023) 11:154. doi: 10.1186/s40168-023-01606-x, PMID: 37468922 PMC10355067

[67] Li Y Cao W Gao NL Zhao XM Chen WH . Consistent alterations of human fecal microbes after transplantation into germ-free mice. Genomics Proteomics Bioinf. (2022) 20:382–93. doi: 10.1016/j.gpb.2020.06.024, PMID: 34118462 PMC9684084

[68] Thaiss CA Levy M Korem T Dohnalova L Shapiro H Jaitin DA . Microbiota diurnal rhythmicity programs host transcriptome oscillations. Cell. (2016) 167:1495–510. doi: 10.1016/j.cell.2016.11.003, PMID: 27912059

[69] Qin J Li R Raes J Arumugam M Burgdorf KS Manichanh C . A human gut microbial gene catalogue established by metagenomic sequencing. Nature. (2010) 464:59–65. doi: 10.1038/nature08821, PMID: 20203603 PMC3779803

[70] Zhang M Zhou C Li X Li H Han Q Chen Z . Interactions between gut microbiota, host circadian rhythms, and metabolic diseases. Adv Nutr. (2025) 16(6):100416. doi: 10.1016/j.advnut.2025.100416, PMID: 40139315 PMC12148639

[71] Liu T Wang Z Ye L Duan Y Jiang H He H . Nucleus-exported clock acetylates prps to promote *de novo* nucleotide synthesis and liver tumour growth. Nat Cell Biol. (2023) 25:273–84. doi: 10.1038/s41556-022-01061-0, PMID: 36646788

[72] Trikha S Lee DM Ecton KE Wrigley SD Vazquez AR Litwin NS . Transplantation of an obesity-associated human gut microbiota to mice induces vascular dysfunction and glucose intolerance. Gut Microbes. (2021) 13:1940791. doi: 10.1080/19490976.2021.1940791, PMID: 34313540 PMC8317959

[73] Chaix A . Time-restricted feeding and caloric restriction: two feeding regimens at the crossroad of metabolic and circadian regulation. Methods Mol Biol. (2022) 2482:329–40. doi: 10.1007/978-1-0716-2249-0_22, PMID: 35610437 PMC9254535

[74] Dantas MA Brown SD Lingaraju A Sivaganesh V Martino C Chaix A . Diet and feeding pattern modulate diurnal dynamics of the ileal microbiome and transcriptome. Cell Rep. (2022) 40:111008. doi: 10.1016/j.celrep.2022.111008, PMID: 35793637 PMC9296000

[75] Noor J Chaudhry A Batool S Noor R Fatima G . Exploring the impact of the gut microbiome on obesity and weight loss: a review article. Cureus. (2023) 15:e40948. doi: 10.7759/cureus.40948, PMID: 37503494 PMC10368799

[76] Pickard JM Zeng MY Caruso R Nunez G . Gut microbiota: role in pathogen colonization, immune responses, and inflammatory disease. Immunol Rev. (2017) 279:70–89. doi: 10.1111/imr.12567, PMID: 28856738 PMC5657496

[77] Yoo JJ Park MY Cho EJ Yu SJ Kim SG Kim YJ . Smoking increases the risk of hepatocellular carcinoma and cardiovascular disease in patients with metabolic-associated fatty liver disease. J Clin Med. (2023) 12:3336. doi: 10.3390/jcm12093336, PMID: 37176776 PMC10179445

[78] Liu Y Dai M Bi Y Xu M Xu Y Li M . Active smoking, passive smoking, and risk of nonalcoholic fatty liver disease (nafld): a population-based study in China. J Epidemiol. (2013) 23:115–21. doi: 10.2188/jea.je20120067, PMID: 23399520 PMC3700247

[79] Li Z Guo H He H Wang S Pei S Xie L . The relationship between smoking cessation history and significant liver fibrosis among the population with metabolic dysfunction-associated steatotic liver disease in the unitedstates. PloS One. (2025) 20:e320573. doi: 10.1371/journal.pone.0320573, PMID: 40168280 PMC11960941

[80] Akhavan RA Dadgar MM Ghasemi NM Shirazinia M Ghodsi H Rouhbakhsh ZM . Association between smoking and non-alcoholic fatty liver disease: a systematic review and meta-analysis. SAGE Open Med. (2018) 6:2104817223. doi: 10.1177/2050312117745223, PMID: 29399359 PMC5788091

[81] Zhou B Gong N He Q Huang X Zhu J Zhang L . Clustering of lifestyle behaviours and analysis of their associations with mafld: a cross-sectional study of 196,515 individuals in China. BMC Public Health. (2023) 23:2303. doi: 10.1186/s12889-023-17177-3, PMID: 37990228 PMC10664514

[82] Gui X Yang Z Li MD . Effect of cigarette smoke on gut microbiota: state of knowledge. Front Physiol. (2021) 12:673341. doi: 10.3389/fphys.2021.673341, PMID: 34220536 PMC8245763

[83] Cicchinelli S Rosa F Manca F Zanza C Ojetti V Covino M . The impact of smoking on microbiota: a narrative review. Biomedicines. (2023) 11:1144. doi: 10.3390/biomedicines11041144, PMID: 37189762 PMC10135766

[84] Mignini I Galasso L Piccirilli G Calvez V Termite F Esposto G . Interplay of oxidative stress, gut microbiota, and nicotine in metabolic-associated steatotic liver disease (masld). Antioxidants (Basel). (2024) 13:1532. doi: 10.3390/antiox13121532, PMID: 39765860 PMC11727446

[85] Soares JB Pimentel-Nunes P Roncon-Albuquerque R Leite-Moreira A . The role of lipopolysaccharide/toll-like receptor 4 signaling in chronic liver diseases. Hepatol Int. (2010) 4:659–72. doi: 10.1007/s12072-010-9219-x, PMID: 21286336 PMC2994611

[86] Shen H Liangpunsakul S Iwakiri Y Szabo G Wang H . Immunological mechanisms and emerging therapeutic targets in alcohol-associated liver disease. Cell Mol Immunol. (2025) 22:1190–204. doi: 10.1038/s41423-025-01291-w, PMID: 40399593 PMC12479882

[87] Ge J Xu WJ Chen HF Dong ZH Liu W Nian FZ . Induction mechanism of cigarette smoke components (cscs) on dyslipidemia and hepatic steatosis in rats. Lipids Health Dis. (2022) 21:117. doi: 10.1186/s12944-022-01725-8, PMID: 36348421 PMC9644616

[88] Wang X Ye P Fang L Ge S Huang F Polverini PJ . Active smoking induces aberrations in digestive tract microbiota of rats. Front Cell Infect Microbiol. (2021) 11:737204. doi: 10.3389/fcimb.2021.737204, PMID: 34917518 PMC8668415

[89] Guo B Huang S Li S Han X Lin H Li Y . Long-term exposure to ambient pm2.5 and its constituents is associated with mafld. JHEP Rep. (2023) 5:100912. doi: 10.1016/j.jhepr.2023.100912, PMID: 37954486 PMC10632732

[90] Wang JT Hu W Xue Z Cai X Zhang SY Li FQ . Mapping multi-omics characteristics related to short-term pm(2.5) trajectory and their impact on type 2 diabetes in middle-aged and elderly adults in southern China. J Hazard Mater. (2024) 468:133784. doi: 10.1016/j.jhazmat.2024.133784, PMID: 38382338

[91] Filardo S Di Pietro M Protano C Antonucci A Vitali M Sessa R . Impact of air pollution on the composition and diversity of human gut microbiota in general and vulnerable populations: a systematic review. Toxics. (2022) 10:579. doi: 10.3390/toxics10100579, PMID: 36287859 PMC9607944

[92] Reyes-Caballero H Rao X Sun Q Warmoes MO Lin P Sussan TE . Air pollution-derived particulate matter dysregulates hepatic krebs cycle, glucose and lipid metabolism in mice. Sci Rep. (2019) 9:17423. doi: 10.1038/s41598-019-53716-y, PMID: 31757983 PMC6874681

[93] Jin X Xue B Zhou Q Su R Li Z . Mitochondrial damage mediated by ros incurs bronchial epithelial cell apoptosis upon ambient pm (2.5) exposure. J Toxicol Sci. (2018) 43:101–11. doi: 10.2131/jts.43.101, PMID: 29479032

[94] Imhann F Bonder MJ Vich VA Fu J Mujagic Z Vork L . Proton pump inhibitors affect the gut microbiome. Gut. (2016) 65:740–8. doi: 10.1136/gutjnl-2015-310376, PMID: 26657899 PMC4853569

[95] Abubucker S Segata N Goll J Schubert AM Izard J Cantarel BL . Metabolic reconstruction for metagenomic data and its application to the human microbiome. PloS Comput Biol. (2012) 8:e1002358. doi: 10.1371/journal.pcbi.1002358, PMID: 22719234 PMC3374609

[96] Fine RL Manfredo VS Gilmore MS Kriegel MA . Mechanisms and consequences of gut commensal translocation in chronic diseases. Gut Microbes. (2020) 11:217–30. doi: 10.1080/19490976.2019.1629236, PMID: 31306081 PMC7053960

[97] Pavlo P Kamyshna I Kamyshnyi A . Effects of metformin on the gut microbiota: a systematic review. Mol Metab. (2023) 77:101805. doi: 10.1016/j.molmet.2023.101805, PMID: 37696355 PMC10518565

[98] Bahne E Sun E Young RL Hansen M Sonne DP Hansen JS . Metformin-induced glucagon-like peptide-1 secretion contributes to the actions of metformin in type 2 diabetes. JCI Insight. (2018) 3:e93936. doi: 10.1172/jci.insight.93936, PMID: 30518693 PMC6328020

[99] Duller S Moissl-Eichinger C . Archaea in the human microbiome and potential effects on human infectious disease. Emerg Infect Dis. (2024) 30:1505–13. doi: 10.3201/eid3008.240181, PMID: 39043386 PMC11286065

[100] Lee CB Chae SU Jo SJ Jerng UM Bae SK . The relationship between the gut microbiome and metformin as a key for treating type 2 diabetes mellitus. Int J Mol Sci. (2021) 22:3566. doi: 10.3390/ijms22073566, PMID: 33808194 PMC8037857

[101] Turunen J Tejesvi MV Paalanne N Pokka T Amatya SB Mishra S . Investigating prenatal and perinatal factors on meconium microbiota: a systematic review and cohort study. Pediatr Res. (2024) 95:135–45. doi: 10.1038/s41390-023-02783-z, PMID: 37591927 PMC10798900

[102] Xie Z Chen Z Chai Y Yao W Ma G . Unveiling the placental bacterial microbiota: implications for maternal and infant health. Front Physiol. (2025) 16:1544216. doi: 10.3389/fphys.2025.1544216, PMID: 40161970 PMC11949977

[103] Lai C Huang L Wang Y Huang C Luo Y Qin X . Effect of different delivery modes on intestinal microbiota and immune function of neonates. Sci Rep. (2024) 14:17452. doi: 10.1038/s41598-024-68599-x, PMID: 39075163 PMC11286838

[104] Derrien M Alvarez AS de Vos WM . The gut microbiota in the first decade of life. Trends Microbiol. (2019) 27:997–1010. doi: 10.1016/j.tim.2019.08.001, PMID: 31474424

[105] Zhang P Zheng L Duan Y Gao Y Gao H Mao D . Gut microbiota exaggerates triclosan-induced liver injury via gut-liver axis. J Hazard Mater. (2022) 421:126707. doi: 10.1016/j.jhazmat.2021.126707, PMID: 34315018

[106] Wu W Kaicen W Bian X Yang L Ding S Li Y . Akkermansia muciniphila alleviates high-fat-diet-related metabolic-associated fatty liver disease by modulating gut microbiota and bile acids. Microb Biotechnol. (2023) 16:1924–39. doi: 10.1111/1751-7915.14293, PMID: 37377410 PMC10527187

[107] Liu J Liu Y Huang C He C Yang T Ren R . Quercetin-driven akkermansia muciniphila alleviates obesity by modulating bile acid metabolism via an ila/m(6)a/cyp8b1 signaling. Adv Sci (Weinh). (2025) 12:e2412865. doi: 10.1002/advs.202412865, PMID: 39888270 PMC11948036

[108] Vicerra P . Population ageing in lower and middle-income countries: policy landscape of southeast asian countries. Curr Aging Sci. (2023) 16:188–93. doi: 10.2174/1874609816666230516150701, PMID: 37194225

[109] Xu C Zhu H Qiu P . Aging progression of human gut microbiota. BMC Microbiol. (2019) 19:236. doi: 10.1186/s12866-019-1616-2, PMID: 31660868 PMC6819604

[110] Escudero-Bautista S Omana-Covarrubias A Nez-Castro AT Lopez-Pontigo L Pimentel-Perez M Chavez-Mejia A . Impact of gut microbiota on aging and frailty: a narrative review of the literature. Geriatrics (Basel). (2024) 9:110. doi: 10.3390/geriatrics9050110, PMID: 39311235 PMC11417718

[111] Li T Apte U . Bile acid metabolism and signaling in cholestasis, inflammation, and cancer. Adv Pharmacol. (2015) 74:263–302. doi: 10.1016/bs.apha.2015.04.003, PMID: 26233910 PMC4615692

[112] Wu SE Hashimoto-Hill S Woo V Eshleman EM Whitt J Engleman L . Microbiota-derived metabolite promotes hdac3 activity in the gut. Nature. (2020) 586:108–12. doi: 10.1038/s41586-020-2604-2, PMID: 32731255 PMC7529926

[113] Woo V Alenghat T . Epigenetic regulation by gut microbiota. Gut Microbes. (2022) 14:2022407. doi: 10.1080/19490976.2021.2022407, PMID: 35000562 PMC8744890

[114] Kulkarni SR Soroka CJ Hagey LR Boyer JL . Sirtuin 1 activation alleviates cholestatic liver injury in a cholic acid-fed mouse model of cholestasis. Hepatology. (2016) 64:2151–64. doi: 10.1002/hep.28826, PMID: 27639250 PMC5115990

[115] Ding RB Bao J Deng CX . Emerging roles of sirt1 in fatty liver diseases. Int J Biol Sci. (2017) 13:852–67. doi: 10.7150/ijbs.19370, PMID: 28808418 PMC5555103

[116] Ahmad R Kumar B Thapa I Talmon GA Salomon J Ramer-Tait AE . Loss of claudin-3 expression increases colitis risk by promoting gut dysbiosis. Gut Microbes. (2023) 15:2282789. doi: 10.1080/19490976.2023.2282789, PMID: 38010872 PMC10730149

[117] Ohguchi Y Ohguchi H . Diverse functions of kdm5 in cancer: transcriptional repressor or activator? Cancers (Basel). (2022) 14:3270. doi: 10.3390/cancers14133270, PMID: 35805040 PMC9265395

[118] Luo L Li Q Xing C Li C Pan Y Sun H . Antibody-based therapy: an alternative for antimicrobial treatment in the post-antibiotic era. Microbiol Res. (2025) 290:127974. doi: 10.1016/j.micres.2024.127974, PMID: 39577369

[119] Zhong XZ Deng Y Chen G Yang H . Investigation of the clinical significance and molecular mechanism of mir-21-5p in hepatocellular carcinoma: a systematic review based on 24 studies and bioinformatics investigation. Oncol Lett. (2019) 17:230–46. doi: 10.3892/ol.2018.9627, PMID: 30655760 PMC6313181

[120] Lu X Chen B Xu D Hu W Wang X Dai Y . Epigenetic programming mediates abnormal gut microbiota and disease susceptibility in offspring with prenatal dexamethasone exposure. Cell Rep Med. (2024) 5:101398. doi: 10.1016/j.xcrm.2024.101398, PMID: 38301654 PMC10897547

[121] Wang JZ Cao HX Chen JN Pan Q . Pnpla3 rs738409 underlies treatment response in nonalcoholic fatty liver disease. World J Clin Cases. (2018) 6:167–75. doi: 10.12998/wjcc.v6.i8.167, PMID: 30148144 PMC6107533

[122] Tikhomirova AS Kislyakov VA Baykova IE Nikitin IG . Clinical-morphological parallels of the pnpla3 gene polymorphism in patients with nonalcoholic fatty liver disease. Ter Arkh. (2018) 90:85–8. doi: 10.26442/terarkh201890285-88, PMID: 30701779

[123] Li XY Liu Z Li L Wang HJ Wang H . Tm6sf2 rs58542926 is related to hepatic steatosis, fibrosis and serum lipids both in adults and children: a meta-analysis. Front Endocrinol (Lausanne). (2022) 13:1026901. doi: 10.3389/fendo.2022.1026901, PMID: 36353245 PMC9637980

[124] Singh N Gurav A Sivaprakasam S Brady E Padia R Shi H . Activation of gpr109a, receptor for niacin and the commensal metabolite butyrate, suppresses colonic inflammation and carcinogenesis. Immunity. (2014) 40:128–39. doi: 10.1016/j.immuni.2013.12.007, PMID: 24412617 PMC4305274

[125] Jacob N Jaiswal S Maheshwari D Nallabelli N Khatri N Bhatia A . Butyrate induced tregs are capable of migration from the galt to the pancreas to restore immunological tolerance during type-1 diabetes. Sci Rep. (2020) 10:19120. doi: 10.1038/s41598-020-76109-y, PMID: 33154424 PMC7644709

[126] Gasaly N Hermoso MA Gotteland M . Butyrate and the fine-tuning of colonic homeostasis: implication for inflammatory bowel diseases. Int J Mol Sci. (2021) 22:3061. doi: 10.3390/ijms22063061, PMID: 33802759 PMC8002420

[127] Du K Bereswill S Heimesaat MM . A literature survey on antimicrobial and immune-modulatory effects of butyrate revealing non-antibiotic approaches to tackle bacterial infections. Eur J Microbiol Immunol (Bp). (2021) 11:1–9. doi: 10.1556/1886.2021.00001, PMID: 33735105 PMC8042652

[128] Prins GH Rios-Morales M Gerding A Reijngoud DJ Olinga P Bakker BM . The effects of butyrate on induced metabolic-associated fatty liver disease in precision-cut liver slices. Nutrients. (2021) 13:4203. doi: 10.3390/nu13124203, PMID: 34959755 PMC8703944

[129] Theofilis P Vordoni A Kalaitzidis RG . Metabolic dysfunction-associated fatty liver disease in the national health and nutrition examination survey 2017-2020: epidemiology, clinical correlates, and the role of diagnostic scores. Metabolites. (2022) 12:1070. doi: 10.3390/metabo12111070, PMID: 36355156 PMC9697527

[130] Chen SN Tan Y Xiao XC Li Q Wu Q Peng YY . Deletion of tlr4 attenuates lipopolysaccharide-induced acute liver injury by inhibiting inflammation and apoptosis. Acta Pharmacol Sin. (2021) 42:1610–9. doi: 10.1038/s41401-020-00597-x, PMID: 33495514 PMC8463538

[131] Zhang Q Shen X Yuan X Huang J Zhu Y Zhu T . Lipopolysaccharide binding protein resists hepatic oxidative stress by regulating lipid droplet homeostasis. Nat Commun. (2024) 15:3213. doi: 10.1038/s41467-024-47553-5, PMID: 38615060 PMC11016120

[132] Zhu YL Meng LL Ma JH Yuan X Chen SW Yi XR . Loss of lbp triggers lipid metabolic disorder through h3k27 acetylation-mediated c/ebpbeta- scd activation in non-alcoholic fatty liver disease. Zool Res. (2024) 45:79–94. doi: 10.24272/j.issn.2095-8137.2023.022, PMID: 38114435 PMC10839665

[133] Ciesielska A Matyjek M Kwiatkowska K . Tlr4 and cd14 trafficking and its influence on lps-induced pro-inflammatory signaling. Cell Mol Life Sci. (2021) 78:1233–61. doi: 10.1007/s00018-020-03656-y, PMID: 33057840 PMC7904555

[134] Nighot M Al-Sadi R Guo S Rawat M Nighot P Watterson MD . Lipopolysaccharide-induced increase in intestinal epithelial tight permeability is mediated by toll-like receptor 4/myeloid differentiation primary response 88 (myd88) activation of myosin light chain kinase expression. Am J Pathol. (2017) 187:2698–710. doi: 10.1016/j.ajpath.2017.08.005, PMID: 29157665 PMC5718096

[135] Chen J Deng X Liu Y Tan Q Huang G Che Q . Kupffer cells in non-alcoholic fatty liver disease: friend or foe? Int J Biol Sci. (2020) 16:2367–78. doi: 10.7150/ijbs.47143, PMID: 32760204 PMC7378652

[136] Fukui H . Role of gut dysbiosis in liver diseases: what have we learned so far? Diseases. (2019) 7:58. doi: 10.3390/diseases7040058, PMID: 31726747 PMC6956030

[137] Crump GM Zhou J Mashayekh S Grimes CL . Revisiting peptidoglycan sensing: interactions with host immunity and beyond. Chem Commun (Camb). (2020) 56:13313–22. doi: 10.1039/d0cc02605k, PMID: 33057506 PMC7642115

[138] Boltana S Reyes-Lopez F Morera D Goetz F Mackenzie SA . Divergent responses to peptidoglycans derived from different e. Coli serotypes influence inflammatory outcome in trout, oncorhynchus mykiss, macrophages. BMC Genomics. (2011) 12:34. doi: 10.1186/1471-2164-12-34, PMID: 21235753 PMC3087353

[139] Humann J Lenz LL . Bacterial peptidoglycan degrading enzymes and their impact on host muropeptide detection. J Innate Immun. (2009) 1:88–97. doi: 10.1159/000181181, PMID: 19319201 PMC2659621

[140] Guo Z Zhang Y Liu C Youn JY Cai H . Toll-like receptor 2 (tlr2) knockout abrogates diabetic and obese phenotypes while restoring endothelial function via inhibition of nox1. Diabetes. (2021) 70:2107–19. doi: 10.2337/db20-0591, PMID: 34127487 PMC8576422

[141] Deng M Qu F Chen L Liu C Zhang M Ren F . Scfas alleviated steatosis and inflammation in mice with nash induced by mcd. J Endocrinol. (2020) 245:425–37. doi: 10.1530/JOE-20-0018, PMID: 32302970

[142] Li Z Shang D . Nod1 and nod2: essential monitoring partners in the innate immune system. Curr Issues Mol Biol. (2024) 46:9463–79. doi: 10.3390/cimb46090561, PMID: 39329913 PMC11430502

[143] Trindade BC Chen GY . Nod1 and nod2 in inflammatory and infectious diseases. Immunol Rev. (2020) 297:139–61. doi: 10.1111/imr.12902, PMID: 32677123 PMC8928416

[144] Chen Z Behrendt R Wild L Schlee M Bode C . Cytosolic nucleic acid sensing as driver of critical illness: mechanisms and advances in therapy. Signal Transduct Target Ther. (2025) 10:90. doi: 10.1038/s41392-025-02174-2, PMID: 40102400 PMC11920230

[145] Giridharan S Srinivasan M . Mechanisms of nf-kappab p65 and strategies for therapeutic manipulation. J Inflammation Res. (2018) 11:407–19. doi: 10.2147/JIR.S140188, PMID: 30464573 PMC6217131

[146] Minton K . Lc3 anchors tlr9 signalling. Nat Rev Immunol. (2018) 18:418–9. doi: 10.1038/s41577-018-0019-1, PMID: 29752467

[147] Csak T Pillai A Ganz M Lippai D Petrasek J Park JK . Both bone marrow-derived and non-bone marrow-derived cells contribute to aim2 and nlrp3 inflammasome activation in a myd88-dependent manner in dietary steatohepatitis. Liver Int. (2014) 34:1402–13. doi: 10.1111/liv.12537, PMID: 24650018 PMC4169310

[148] Garcia-Martinez I Santoro N Chen Y Hoque R Ouyang X Caprio S . Hepatocyte mitochondrial dna drives nonalcoholic steatohepatitis by activation of tlr9. J Clin Invest. (2016) 126:859–64. doi: 10.1172/JCI83885, PMID: 26808498 PMC4767345

[149] Jackson SW Scharping NE Kolhatkar NS Khim S Schwartz MA Li QZ . Opposing impact of b cell-intrinsic tlr7 and tlr9 signals on autoantibody repertoire and systemic inflammation. J Immunol. (2014) 192:4525–32. doi: 10.4049/jimmunol.1400098, PMID: 24711620 PMC4041708

[150] Ma X Huang T Chen X Li Q Liao M Fu L . Molecular mechanisms in liver repair and regeneration: from physiology to therapeutics. Signal Transduct Target Ther. (2025) 10:63. doi: 10.1038/s41392-024-02104-8, PMID: 39920130 PMC11806117

[151] Qu W Ma T Cai J Zhang X Zhang P She Z . Liver fibrosis and mafld: from molecular aspects to novel pharmacological strategies. Front Med (Lausanne). (2021) 8:761538. doi: 10.3389/fmed.2021.761538, PMID: 34746195 PMC8568774

[152] Furuyama N Sircili MP . Outer membrane vesicles (omvs) produced by gram-negative bacteria: structure, functions, biogenesis, and vaccine application. BioMed Res Int. (2021) 2021:1490732. doi: 10.1155/2021/1490732, PMID: 33834062 PMC8016564

[153] Vanaja SK Russo AJ Behl B Banerjee I Yankova M Deshmukh SD . Bacterial outer membrane vesicles mediate cytosolic localization of lps and caspase-11 activation. Cell. (2016) 165:1106–19. doi: 10.1016/j.cell.2016.04.015, PMID: 27156449 PMC4874922

[154] Yi YS . Caspase-11 non-canonical inflammasome: a critical sensor of intracellular lipopolysaccharide in macrophage-mediated inflammatory responses. Immunology. (2017) 152:207–17. doi: 10.1111/imm.12787, PMID: 28695629 PMC5588777

[155] Chen S Lei Q Zou X Ma D . The role and mechanisms of gram-negative bacterial outer membrane vesicles in inflammatory diseases. Front Immunol. (2023) 14:1157813. doi: 10.3389/fimmu.2023.1157813, PMID: 37398647 PMC10313905

[156] Yang K Song M . New insights into the pathogenesis of metabolic-associated fatty liver disease (mafld): gut-liver-heart crosstalk. Nutrients. (2023) 15:3970. doi: 10.3390/nu15183970, PMID: 37764755 PMC10534946

[157] Cong J Zhou P Zhang R . Intestinal microbiota-derived short chain fatty acids in host health and disease. Nutrients. (2022) 14:1977. doi: 10.3390/nu14091977, PMID: 35565943 PMC9105144

[158] Kimura I Inoue D Hirano K Tsujimoto G . The scfa receptor gpr43 and energy metabolism. Front Endocrinol (Lausanne). (2014) 5:85. doi: 10.3389/fendo.2014.00085, PMID: 24926285 PMC4046487

[159] Feng Y Xu D . Short-chain fatty acids are potential goalkeepers of atherosclerosis. Front Pharmacol. (2023) 14:1271001. doi: 10.3389/fphar.2023.1271001, PMID: 38027009 PMC10679725

[160] Boini KM Hussain T Li PL Koka S . Trimethylamine-n-oxide instigates nlrp3 inflammasome activation and endothelial dysfunction. Cell Physiol Biochem. (2017) 44:152–62. doi: 10.1159/000484623, PMID: 29130962 PMC5828122

[161] Watanabe M Houten SM Wang L Moschetta A Mangelsdorf DJ Heyman RA . Bile acids lower triglyceride levels via a pathway involving fxr, shp, and srebp-1c. J Clin Invest. (2004) 113:1408–18. doi: 10.1172/JCI21025, PMID: 15146238 PMC406532

[162] Kumari A Pal PD Asthana S . Bile acids mediated potential functional interaction between fxr and fatp5 in the regulation of lipid metabolism. Int J Biol Sci. (2020) 16:2308–22. doi: 10.7150/ijbs.44774, PMID: 32760200 PMC7378638

[163] Segura-Azuara N Varela-Chinchilla CD Trinidad-Calderon PA . Mafld/nafld biopsy-free scoring systems for hepatic steatosis, nash, and fibrosis diagnosis. Front Med (Lausanne). (2021) 8:774079. doi: 10.3389/fmed.2021.774079, PMID: 35096868 PMC8792949

[164] Nogal A Valdes AM Menni C . The role of short-chain fatty acids in the interplay between gut microbiota and diet in cardio-metabolic health. Gut Microbes. (2021) 13:1–24. doi: 10.1080/19490976.2021.1897212, PMID: 33764858 PMC8007165

[165] Richards P Pais R Habib AM Brighton CA Yeo GS Reimann F . High fat diet impairs the function of glucagon-like peptide-1 producing l-cells. Peptides. (2016) 77:21–7. doi: 10.1016/j.peptides.2015.06.006, PMID: 26145551 PMC4788507

[166] Herzig S Shaw RJ . Ampk: guardian of metabolism and mitochondrial homeostasis. Nat Rev Mol Cell Biol. (2018) 19:121–35. doi: 10.1038/nrm.2017.95, PMID: 28974774 PMC5780224

[167] Gao X Lin SH Ren F Li JT Chen JJ Yao CB . Acetate functions as an epigenetic metabolite to promote lipid synthesis under hypoxia. Nat Commun. (2016) 7:11960. doi: 10.1038/ncomms11960, PMID: 27357947 PMC4931325

[168] Arora T Tremaroli V . Therapeutic potential of butyrate for treatment of type 2 diabetes. Front Endocrinol (Lausanne). (2021) 12:761834. doi: 10.3389/fendo.2021.761834, PMID: 34737725 PMC8560891

[169] Thing M Werge MP Kimer N Hetland LE Rashu EB Nabilou P . Targeted metabolomics reveals plasma short-chain fatty acids are associated with metabolic dysfunction-associated steatotic liver disease. BMC Gastroenterol. (2024) 24:43. doi: 10.1186/s12876-024-03129-7, PMID: 38262952 PMC10804800

[170] Rizzolo D Buckley K Kong B Zhan L Shen J Stofan M . Bile acid homeostasis in a cholesterol 7alpha-hydroxylase and sterol 27-hydroxylase double knockout mouse model. Hepatology. (2019) 70:389–402. doi: 10.1002/hep.30612, PMID: 30864232 PMC7893641

[171] Lai J Luo L Zhou T Feng X Ye J Zhong B . Alterations in circulating bile acids in metabolic dysfunction-associated steatotic liver disease: a systematic review and meta-analysis. Biomolecules. (2023) 13:1356. doi: 10.3390/biom13091356, PMID: 37759756 PMC10526305

[172] Chiang JY . Negative feedback regulation of bile acid metabolism: impact on liver metabolism and diseases. Hepatology. (2015) 62:1315–7. doi: 10.1002/hep.27964, PMID: 26122550 PMC4589461

[173] Rao J Yang C Yang S Lu H Hu Y Lu L . Deficiency of tgr5 exacerbates immune-mediated cholestatic hepatic injury by stabilizing the beta-catenin destruction complex. Int Immunol. (2020) 32:321–34. doi: 10.1093/intimm/dxaa002, PMID: 31930324 PMC7206975

[174] Hu X Yan J Huang L Araujo C Peng J Gao L . Int-777 attenuates nlrp3-asc inflammasome-mediated neuroinflammation via tgr5/camp/pka signaling pathway after subarachnoid hemorrhage in rats. Brain Behav Immun. (2021) 91:587–600. doi: 10.1016/j.bbi.2020.09.016, PMID: 32961266 PMC7749833

[175] Pockros PJ Fuchs M Freilich B Schiff E Kohli A Lawitz EJ . Control: a randomized phase 2 study of obeticholic acid and atorvastatin on lipoproteins in nonalcoholic steatohepatitis patients. Liver Int. (2019) 39:2082–93. doi: 10.1111/liv.14209, PMID: 31402538

[176] Cai YY Huang FQ Lao X Lu Y Gao X Alolga RN . Integrated metagenomics identifies a crucial role for trimethylamine-producing lachnoclostridium in promoting atherosclerosis. NPJ Biofilms Microbiomes. (2022) 8:11. doi: 10.1038/s41522-022-00273-4, PMID: 35273169 PMC8913745

[177] Li X Hong J Wang Y Pei M Wang L Gong Z . Trimethylamine-n-oxide pathway: a potential target for the treatment of mafld. Front Mol Biosci. (2021) 8:733507. doi: 10.3389/fmolb.2021.733507, PMID: 34660695 PMC8517136

[178] Yoo W Zieba JK Foegeding NJ Torres TP Shelton CD Shealy NG . High-fat diet-induced colonocyte dysfunction escalates microbiota-derived trimethylamine n-oxide. Science. (2021) 373:813–8. doi: 10.1126/science.aba3683, PMID: 34385401 PMC8506909

[179] Yellaturu CR Deng X Park EA Raghow R Elam MB . Insulin enhances the biogenesis of nuclear sterol regulatory element-binding protein (srebp)-1c by posttranscriptional down-regulation of insig-2a and its dissociation from srebp cleavage-activating protein (scap). srebp-1c complex. J Biol Chem. (2009) 284:31726–34. doi: 10.1074/jbc.M109.050914, PMID: 19759400 PMC2797243

[180] Hu J Zheng Z Lei J Cao Y Li Q Zheng Z . Targeting the ezh2-ppar axis is a potential therapeutic pathway for pancreatic cancer. PPAR Res. (2021) 2021:5589342. doi: 10.1155/2021/5589342, PMID: 34335707 PMC8321753

[181] Mirji G Worth A Bhat SA El SM Kannan T Goldman AR . The microbiome-derived metabolite tmao drives immune activation and boosts responsesto immune checkpoint blockade in pancreatic cancer. Sci Immunol. (2022) 7:eabn704. doi: 10.1126/sciimmunol.abn0704, PMID: 36083892 PMC9925043

[182] Li Y Wang L Yi Q Luo L Xiong Y . Regulation of bile acids and their receptor fxr in metabolic diseases. Front Nutr. (2024) 11:1447878. doi: 10.3389/fnut.2024.1447878, PMID: 39726876 PMC11669848

[183] Artlett CM . The mechanism and regulation of the nlrp3 inflammasome during fibrosis. Biomolecules. (2022) 12:634. doi: 10.3390/biom12050634, PMID: 35625564 PMC9138796

[184] Cole LK Vance DE . A role for sp1 in transcriptional regulation of phosphatidylethanolamine n-methyltransferase in liver and 3t3-l1 adipocytes. J Biol Chem. (2010) 285:11880–91. doi: 10.1074/jbc.M110.109843, PMID: 20150657 PMC2852925

[185] Tamura R Sabu Y Mizuno T Mizuno S Nakano S Suzuki M . Intestinal atp8b1 dysfunction causes hepatic choline deficiency and steatohepatitis. Nat Commun. (2023) 14:6763. doi: 10.1038/s41467-023-42424-x, PMID: 37990006 PMC10663612

[186] Arias N Arboleya S Allison J Kaliszewska A Higarza SG Gueimonde M . The relationship between choline bioavailability from diet, intestinal microbiota composition, and its modulation of human diseases. Nutrients. (2020) 12:2340. doi: 10.3390/nu12082340, PMID: 32764281 PMC7468957

[187] Dinicolantonio JJ Mccarty M Okeefe J . Association of moderately elevated trimethylamine n-oxide with cardiovascular risk: is tmao serving as a marker for hepatic insulin resistance. Open Heart. (2019) 6:e890. doi: 10.1136/openhrt-2018-000890, PMID: 30997120 PMC6443140

[188] Guo Z Zeng C Shen Y Hu L Zhang H Li Z . Helper lipid-enhanced mrna delivery for treating metabolic dysfunction-associated fatty liver disease. Nano Lett. (2024) 24:6743–52. doi: 10.1021/acs.nanolett.4c01458, PMID: 38783628

[189] Meijnikman AS Nieuwdorp M Schnabl B . Endogenous ethanol production in health and disease. Nat Rev Gastroenterol Hepatol. (2024) 21:556–71. doi: 10.1038/s41575-024-00937-w, PMID: 38831008 PMC13742178

[190] Meijnikman AS Davids M Herrema H Aydin O Tremaroli V Rios-Morales M . Microbiome-derived ethanol in nonalcoholic fatty liver disease. Nat Med. (2022) 28:2100–6. doi: 10.1038/s41591-022-02016-6, PMID: 36216942

[191] Li N Li X Ding Y Liu X Diggle K Kisseleva T . Srebp regulation of lipid metabolism in liver disease, and therapeutic strategies. Biomedicines. (2023) 11:3280. doi: 10.3390/biomedicines11123280, PMID: 38137501 PMC10740981

[192] Warren JS Oka SI Zablocki D Sadoshima J . Metabolic reprogramming via pparalpha signaling in cardiac hypertrophy and failure: from metabolomics to epigenetics. Am J Physiol Heart Circ Physiol. (2017) 313:H584–96. doi: 10.1152/ajpheart.00103.2017, PMID: 28646024 PMC6425516

[193] Elamin E Jonkers D Juuti-Uusitalo K van Ijzendoorn S Troost F Duimel H . Effects of ethanol and acetaldehyde on tight junction integrity: *in vitro* study in a threedimensional intestinal epithelial cell culture model. PloS One. (2012) 7:e35008. doi: 10.1371/journal.pone.0035008, PMID: 22563376 PMC3339854

[194] Chong PL Laight D Aspinall RJ Higginson A Cummings MH . A randomised placebocontrolled trial of vsl3((r)) probiotic on biomarkers of cardiovascular risk and liver injury in non-alcoholic fatty liver disease. BMC Gastroenterol. (2021) 21:144. doi: 10.1186/s12876-021-01660-5, PMID: 33794784 PMC8015038

[195] Jena PK Sheng L Li Y Wan YY . Probiotics vsl3 are effective in reversing non-alcoholic steatohepatitis in a mouse model. Hepatobiliary Surg Nutr. (2020) 9:170–82. doi: 10.21037/hbsn.2019.09.07, PMID: 32355675 PMC7188546

[196] Alisi A Mccaughan G Gronbaek H . Role of gut microbiota and immune cells in metabolic-associated fatty liver disease: clinical impact. Hepatol Int. (2024) 18:861–72. doi: 10.1007/s12072-024-10674-6, PMID: 38995341

[197] Kang Y Su G Sun J Zhang Y . Activation of the tlr4/myd88 signaling pathway contributes to the development of human hepatocellular carcinoma via upregulation of il-23 and il-17a. Oncol Lett. (2018) 15:9647–54. doi: 10.3892/ol.2018.8586, PMID: 29928340 PMC6004652

[198] Blevins HM Xu Y Biby S Zhang S . The nlrp3 inflammasome pathway: a review of mechanisms and inhibitors for the treatment of inflammatory diseases. Front Aging Neurosci. (2022) 14:879021. doi: 10.3389/fnagi.2022.879021, PMID: 35754962 PMC9226403

[199] Frampton J Murphy KG Frost G Chambers ES . Short-chain fatty acids as potential regulators of skeletal muscle metabolism and function. Nat Metab. (2020) 2:840–8. doi: 10.1038/s42255-020-0188-7, PMID: 32694821

[200] Han YH Onufer EJ Huang LH Sprung RW Davidson WS Czepielewski RS . Enterically derived high-density lipoprotein restrains liver injury through the portal vein. Science. (2021) 373:6729. doi: 10.1126/science.Abe6729, PMID: 34437091 PMC8478306

[201] Wang K . Molecular mechanisms of hepatic apoptosis. Cell Death Dis. (2014) 5:e996. doi: 10.1038/cddis.2013.499, PMID: 24434519 PMC4040708

[202] Stasi A Fiorentino M Franzin R Staffieri F Carparelli S Losapio R . Beneficial effects of recombinant cer-001 high-density lipoprotein infusion in sepsis: results from a bench to bedside translational research project. BMC Med. (2023) 21:392. doi: 10.1186/s12916-023-03057-5, PMID: 37915050 PMC10621167

[203] Massier L Bluher M Kovacs P Chakaroun RM . Impaired intestinal barrier and tissue bacteria: pathomechanisms for metabolic diseases. Front Endocrinol (Lausanne). (2021) 12:616506. doi: 10.3389/fendo.2021.616506, PMID: 33767669 PMC7985551

[204] Sakai J Cammarota E Wright JA Cicuta P Gottschalk RA Li N . Lipopolysaccharide-induced nf-kappab nuclear translocation is primarily dependent on myd88, but tnfalpha expression requires trif and myd88. Sci Rep. (2017) 7:1428. doi: 10.1038/s41598-017-01600-y, PMID: 28469251 PMC5431130

[205] Liang H Hussey SE Sanchez-Avila A Tantiwong P Musi N . Effect of lipopolysaccharide on inflammation and insulin action in human muscle. PloS One. (2013) 8:e63983. doi: 10.1371/journal.pone.0063983, PMID: 23704966 PMC3660322

[206] Pezzino S Sofia M Faletra G Mazzone C Litrico G La Greca G . Gut–liver axis and non-alcoholic fatty liver disease: A vicious circle of dysfunctions orchestrated by the gut microbiome. Biol (Basel). (2022) 11:1622. doi: 10.3390/biology11111622, PMID: 36358323 PMC9687983

[207] Joon A Sharma A Jalandra R Bayal N Dhar R Karmakar S . Nonalcoholic fatty liver disease and gut-liver axis: role of intestinal microbiota and therapeutic mechanisms. J Trans Gastroenterology. (2024) 2:38–51. doi: 10.14218/JTG.2023.00018

[208] Tarantino G Citro V Balsano C . Liver-spleen axis in nonalcoholic fatty liver disease. Expert Rev Gastroenterol Hepatol. (2021) 15:759–69. doi: 10.1080/17474124.2021.1914587, PMID: 33878988

[209] Fu Y Gong T Loughran PA Li Y Billiar TR Liu Y . Roles of tlr4 in macrophage immunity and macrophage-pulmonary vascular/lymphatic endothelial cell interactions in sepsis. Commun Biol. (2025) 8:469. doi: 10.1038/s42003-025-07921-3, PMID: 40119011 PMC11928643

[210] Unterberger S Mullen L Flint MS Sacre S . Multiple tlrs elicit alternative nlrp3 inflammasome activation in primary human monocytes independent of ripk1 kinase activity. Front Immunol. (2023) 14:1092799. doi: 10.3389/fimmu.2023.1092799, PMID: 37954581 PMC10639122

[211] Ogawa Y Imajo K Yoneda M Kessoku T Tomeno W Shinohara Y . Soluble cd14 levels reflect liver inflammation in patients with nonalcoholic steatohepatitis. PloS One. (2013) 8:e65211. doi: 10.1371/journal.pone.0065211, PMID: 23762319 PMC3676404

[212] Ono Y Maejima Y Saito M Sakamoto K Horita S Shimomura K . Tak-242, a specific inhibitor of toll-like receptor 4 signalling, prevents endotoxemia-induced skeletal muscle wasting in mice. Sci Rep. (2020) 10:694. doi: 10.1038/s41598-020-57714-3, PMID: 31959927 PMC6970997

[213] Liu S Wu J Chen P Mohammed S Zhang J Liu S . Tak-242 ameliorates hepatic fibrosis by regulating the liver-gut axis. BioMed Res Int. (2022) 2022:4949148. doi: 10.1155/2022/4949148, PMID: 36017390 PMC9398794

[214] Keating SE Sabag A Hallsworth K Hickman IJ Macdonald GA Stine JG . Exercise in the management of metabolic-associated fatty liver disease (mafld) in adults: a position statement from exercise and sport science Australia. Sports Med. (2023) 53:2347–71. doi: 10.1007/s40279-023-01918-w, PMID: 37695493 PMC10687186

[215] Cullen J Shahzad S Dhillon J . A systematic review on the effects of exercise on gut microbial diversity, taxonomic composition, and microbial metabolites: identifying research gaps and future directions. Front Physiol. (2023) 14:1292673. doi: 10.3389/fphys.2023.1292673, PMID: 38187136 PMC10770260

[216] Funabashi M Grove TL Wang M Varma Y Mcfadden ME Brown LC . A metabolic pathway for bile acid dehydroxylation by the gut microbiome. Nature. (2020) 582:566–70. doi: 10.1038/s41586-020-2396-4, PMID: 32555455 PMC7319900

[217] Deng D Xu L Liu Y Li C Jiang Q Shi J . Hiit versus mict in masld: mechanisms mediated by gut-liver axis crosstalk, mitochondrial dynamics remodeling, and adipokine signaling attenuation. Lipids Health Dis. (2025) 24:144. doi: 10.1186/s12944-025-02565-y, PMID: 40241065 PMC12004573

[218] Lensu S Pekkala S . Gut microbiota, microbial metabolites and human physical performance. Metabolites. (2021) 11:716. doi: 10.3390/metabo11110716, PMID: 34822374 PMC8619554

[219] Aja E Zeng A Gray W Connelley K Chaganti A Jacobs JP . Health effects and therapeutic potential of the gut microbe akkermansia muciniphila. Nutrients. (2025) 17:562. doi: 10.3390/nu17030562, PMID: 39940420 PMC11820462

[220] Ye Y Xu H Xie Z Wang L Sun Y Yang H . Time-restricted feeding reduces the detrimental effects of a high-fat diet, possibly by modulating the circadian rhythm of hepatic lipid metabolism and gut microbiota. Front Nutr. (2020) 7:596285. doi: 10.3389/fnut.2020.596285, PMID: 33425971 PMC7793950

[221] Feng R Yang W Feng W Huang X Cen M Peng G . Time-restricted feeding ameliorates non-alcoholic fatty liver disease through modulating hepatic nicotinamide metabolism via gut microbiota remodeling. Gut Microbes. (2024) 16:2390164. doi: 10.1080/19490976.2024.2390164, PMID: 39154362 PMC11332628

[222] Yang Z Su H Lv Y Tao H Jiang Y Ni Z . Inulin intervention attenuates hepatic steatosis in rats via modulating gut microbiota and maintaining intestinal barrier function. Food Res Int. (2023) 163:112309. doi: 10.1016/j.foodres.2022.112309, PMID: 36596207

[223] Hu Y Zhou J Lin X . Akkermansia muciniphila helps in the recovery of lipopolysaccharide-fed mice with mild intestinal dysfunction. Front Microbiol. (2025) 16:1523742. doi: 10.3389/fmicb.2025.1523742, PMID: 40143870 PMC11938125

[224] Duseja A Acharya SK Mehta M Chhabra S Rana S Das A . High potency multistrain probiotic improves liver histology in non-alcoholic fatty liver disease (nafld): a randomised, double-blind, proof of concept study. BMJ Open Gastroenterol. (2019) 6:e315. doi: 10.1136/bmjgast-2019-000315, PMID: 31423319 PMC6688701

[225] Kondo T Kishi M Fushimi T Kaga T . Acetic acid upregulates the expression of genes for fatty acid oxidation enzymes in liver to suppress body fat accumulation. J Agric Food Chem. (2009) 57:5982–6. doi: 10.1021/jf900470c, PMID: 19469536

[226] Takeda T Asaoka D Nojiri S Yanagisawa N Nishizaki Y Osada T . Usefulness of bifidobacterium longum bb536 in elderly individuals with chronic constipation: a randomized controlled trial. Am J Gastroenterol. (2023) 118:561–8. doi: 10.14309/ajg.0000000000002028, PMID: 36216361 PMC9973440

[227] Mills S Yang B Smith GJ Stanton C Ross RP . Efficacy of bifidobacterium longum alone or in multi-strain probiotic formulations during early life and beyond. Gut Microbes. (2023) 15:2186098. doi: 10.1080/19490976.2023.2186098, PMID: 36896934 PMC10012958

[228] Sanders ME Akkermans LM Haller D Hammerman C Heimbach J Hormannsperger G . Safety assessment of probiotics for human use. Gut Microbes. (2010) 1:164–85. doi: 10.4161/gmic.1.3.12127, PMID: 21327023 PMC3023597

[229] Sanders ME Merenstein DJ Ouwehand AC Reid G Salminen S Cabana MD . Probiotic use in at-risk populations. J Am Pharm Assoc (2003). (2016) 56:680–6. doi: 10.1016/j.japh.2016.07.001, PMID: 27836128

[230] Pourghassem GB Dehghan P Aliasgharzadeh A Asghari JM . Effects of highperformance inulin supplementation on glycemic control and antioxidant status in women with type 2 diabetes. Diabetes Metab J. (2013) 37:140–8. doi: 10.4093/dmj.2013.37.2.140, PMID: 23641355 PMC3638225

[231] Makki K Brolin H Petersen N Henricsson M Christensen DP Khan MT . 6alpha-hydroxylated bile acids mediate tgr5 signalling to improve glucose metabolism upon dietary fiber supplementation in mice. Gut. (2023) 72:314–24. doi: 10.1136/gutjnl-2021-326541, PMID: 35697422 PMC9872241

[232] Dai Z Feng S Liu AB Wang H Zeng X Yang CS . Protective effects of alpha-galacto-oligosaccharides against a high-fat/western-style diet-induced metabolic abnormalities in mice. Food Funct. (2019) 10:3660–70. doi: 10.1039/c9fo00463g, PMID: 31166330 PMC6588291

[233] Caussy C Reeder SB Sirlin CB Loomba R . Noninvasive, quantitative assessment of liver fat by mri-pdff as an endpoint in nash trials. Hepatology. (2018) 68:763–72. doi: 10.1002/hep.29797, PMID: 29356032 PMC6054824

[234] Torre A Cordova-Gallardo J Frati MA . Rifaximin alfa and liver diseases: more than a treatment for encephalopathy, a disease modifier. Ther Clin Risk Manage. (2023) 19:839–51. doi: 10.2147/TCRM.S425292, PMID: 37899985 PMC10612522

[235] Jian J Nie MT Xiang B Qian H Yin C Zhang X . Rifaximin ameliorates non-alcoholic steatohepatitis in mice through regulating gut microbiome-related bile acids. Front Pharmacol. (2022) 13:841132. doi: 10.3389/fphar.2022.841132, PMID: 35450049 PMC9017645

[236] Wan YP Li S Li D Huang XM Wu JH Jian J . Study on the molecular mechanisms of rifaximin in the treatment of non−alcoholic steatohepatitis based on the helicobacter−dca−fxr−hnf1alpha signalling pathway. Mol Med Rep. (2025) 31:42. doi: 10.3892/mmr.2024.13407, PMID: 39611479 PMC11632295

[237] Wang JS Liu JC . Intestinal microbiota in the treatment of metabolically associated fatty liver disease. World J Clin Cases. (2022) 10:11240–51. doi: 10.12998/wjcc.v10.i31.11240, PMID: 36387806 PMC9649557

[238] Jayakumar S Loomba R . Review article: emerging role of the gut microbiome in the progression of nonalcoholic fatty liver disease and potential therapeutic implications. Aliment Pharmacol Ther. (2019) 50:144–58. doi: 10.1111/apt.15314, PMID: 31149745 PMC6771496

[239] Sun D Zuo C Huang W Wang J Zhang Z . Triclosan targeting of gut microbiome ameliorates hepatic steatosis in high fat diet-fed mice. J Antibiot (Tokyo). (2022) 75:341–53. doi: 10.1038/s41429-022-00522-w, PMID: 35440769

[240] Vliex L Penders J Nauta A Zoetendal EG Blaak EE . The individual response to antibiotics and diet - insights into gut microbial resilience and host metabolism. Nat Rev Endocrinol. (2024) 20:387–98. doi: 10.1038/s41574-024-00966-0, PMID: 38486011

[241] Mahana D Trent CM Kurtz ZD Bokulich NA Battaglia T Chung J . Antibiotic perturbation of the murine gut microbiome enhances the adiposity, insulin resistance, and liver disease associated with high-fat diet. Genome Med. (2016) 8:48. doi: 10.1186/s13073-016-0297-9, PMID: 27124954 PMC4847194

[242] Zhong W Li Q Zhang W Sun Q Sun X Zhou Z . Modulation of intestinal barrier and bacterial endotoxin production contributes to the beneficial effect of nicotinic acid on alcohol-induced endotoxemia and hepatic inflammation in rats. Biomolecules. (2015) 5:2643–58. doi: 10.3390/biom5042643, PMID: 26501337 PMC4693251

[243] de Groot P Scheithauer T Bakker GJ Prodan A Levin E Khan MT . Donor metabolic characteristics drive effects of faecal microbiota transplantation on recipient insulin sensitivity, energy expenditure and intestinal transit time. Gut. (2020) 69:502–12. doi: 10.1136/gutjnl-2019-318320, PMID: 31147381 PMC7034343

[244] Liu Y Li X Chen Y Yao Q Zhou J Wang X . Fecal microbiota transplantation: application scenarios, efficacy prediction, and factors impacting donor-recipient interplay. Front Microbiol. (2025) 16:1556827. doi: 10.3389/fmicb.2025.1556827, PMID: 40201444 PMC11975908

[245] Noureddin M . Utilization of noninvasive tests to diagnose at-risk metabolic dysfunction-associated steatohepatitis. Gastroenterol Hepatol (N Y). (2023) 19:568–70. PMC10524407 37771796

[246] Craven L Rahman A Nair PS Beaton M Silverman J Qumosani K . Allogenic fecal microbiota transplantation in patients with nonalcoholic fatty liver disease improves abnormal small intestinal permeability: a randomized control trial. Am J Gastroenterol. (2020) 115:1055–65. doi: 10.14309/ajg.0000000000000661, PMID: 32618656

[247] Merrick B Allen L Masirah MZN Forbes B Shawcross DL Goldenberg SD . Regulation, risk and safety of faecal microbiota transplant. Infect Prev Pract. (2020) 2:100069. doi: 10.1016/j.infpip.2020.100069, PMID: 34316559 PMC7280140

[248] Savchenko T Degtyaryov E Radzyukevich Y Buryak V . Therapeutic potential of plant oxylipins. Int J Mol Sci. (2022) 23:14627. doi: 10.3390/ijms232314627, PMID: 36498955 PMC9741157

[249] Yi H Wang L Xiong Y Wang Z Qiu Y Wen X . Lactobacillus reuteri lr1 improved expression of genes of tight junction proteins via the mlck pathway in ipec-1 cells during infection with enterotoxigenic escherichia coli k88. Mediators Inflamm. (2018) 2018:6434910. doi: 10.1155/2018/6434910, PMID: 30210262 PMC6120278

[250] Zhang R Tang Y Feng X Lu X Zhao M Jin J . Targeted modulation of intestinal barrier and mucosal immune-related microbiota attenuates iga nephropathy progression. Gut Microbes. (2025) 17:2458184. doi: 10.1080/19490976.2025.2458184, PMID: 39875350 PMC11776482

[251] Fang M Zhang R Wang C Liu Z Fei M Tang B . Engineering probiotic escherichia coli nissle 1917 to block transfer of multiple antibiotic resistance genes by exploiting a type i crispr-cas system. Appl Environ Microbiol. (2024) 90:e81124. doi: 10.1128/aem.00811-24, PMID: 39254327 PMC11497782

[252] Zhuge A Li S Han S Yuan Y Shen J Wu W . Akkermansia muciniphila-derived acetate activates the hepatic ampk/sirt1/pgc-1alpha axis to alleviate ferroptosis in metabolic-associated fatty liver disease. Acta Pharm Sin B. (2025) 15:151–67. doi: 10.1016/j.apsb.2024.10.010, PMID: 40041901 PMC11873632

[253] Palsson-Mcdermott EM O’Neill L . Gang of 3: how the krebs cycle-linked metabolites itaconate, succinate, and fumarate regulate macrophages and inflammation. Cell Metab. (2025) 37:1049–59. doi: 10.1016/j.cmet.2025.03.004, PMID: 40169002

[254] Duan Y Llorente C Lang S Brandl K Chu H Jiang L . Bacteriophage targeting of gut bacterium attenuates alcoholic liver disease. Nature. (2019) 575:505–11. doi: 10.1038/s41586-019-1742-x, PMID: 31723265 PMC6872939

[255] Zixin Y Lulu C Xiangchang Z Qing F Binjie Z Chunyang L . Tmao as a potential biomarker and therapeutic target for chronic kidney disease: a review. Front Pharmacol. (2022) 13:929262. doi: 10.3389/fphar.2022.929262, PMID: 36034781 PMC9411716

[256] Plaza-Diaz J Solis-Urra P Rodriguez-Rodriguez F Olivares-Arancibia J Navarro-Oliveros M Abadia-Molina F . The gut barrier, intestinal microbiota, and liver disease: molecular mechanisms and strategies to manage. Int J Mol Sci. (2020) 21:8351. doi: 10.3390/ijms21218351, PMID: 33171747 PMC7664383

[257] Hao F Tian M Zhang X Jin X Jiang Y Sun X . Butyrate enhances cpt1a activity to promote fatty acid oxidation and itreg differentiation. Proc Natl Acad Sci U.S.A. (2021) 118:e2014681118. doi: 10.1073/pnas.2014681118, PMID: 34035164 PMC8179238

[258] Ling R Chen G Tang X Liu N Zhou Y Chen D . Acetyl-coa synthetase 2(acss2): a review with a focus on metabolism and tumor development. Discov Oncol. (2022) 13:58. doi: 10.1007/s12672-022-00521-1, PMID: 35798917 PMC9263018

[259] Cheng Z Taylor B Ourthiague DR Hoffmann A . Distinct single-cell signaling characteristics are conferred by the myd88 and trif pathways during tlr4 activation. Sci Signal. (2015) 8:ra69. doi: 10.1126/scisignal.aaa5208, PMID: 26175492 PMC6764925

[260] Jiang Y Tang Y Hoover C Kondo Y Huang D Restagno D . Kupffer cell receptor clec4f is important for the destruction of desialylated platelets in mice. Cell Death Differ. (2021) 28:3009–21. doi: 10.1038/s41418-021-00797-w, PMID: 33993195 PMC8564511

[261] Zhai L Pei H Yang Y Zhu Y Ruan S . Nox4 promotes kupffer cell inflammatory response via ros-nlrp3 to aggravate liver inflammatory injury in acute liver injury. Aging (Albany NY). (2022) 14:6905–16. doi: 10.18632/aging.204173, PMID: 35832027 PMC9512511

[262] Lee JH Kang HS Park HY Moon YA Kang YN Oh BC . Pparalpha-dependent insig2a overexpression inhibits srebp-1c processing during fasting. Sci Rep. (2017) 7:9958. doi: 10.1038/s41598-017-10523-7, PMID: 28855656 PMC5577246

[263] Goguyer-Deschaumes R Waeckel L Killian M Rochereau N Paul S . Metabolites and secretory immunoglobulins: messengers and effectors of the host-microbiota intestinal equilibrium. Trends Immunol. (2022) 43:63–77. doi: 10.1016/j.it.2021.11.005, PMID: 34848167

[264] Wang H Wen C Chen S Li W Qin Q He L . Ros/jnk/c-jun pathway is involved in chaetocin induced colorectal cancer cells apoptosis and macrophage phagocytosis enhancement. Front Pharmacol. (2021) 12:729367. doi: 10.3389/fphar.2021.729367, PMID: 34776955 PMC8578663

[265] Shivatare SS Shivatare VS Wong CH . Correction to glycoconjugates: synthesis, functional studies, and therapeutic developments. Chem Rev. (2024) 124:6693–6. doi: 10.1021/acs.chemrev.4c00257, PMID: 38723596

[266] Hu EA Lazo M Selvin E Hamilton JP Grams ME Steffen LM . Coffee consumption and liver-related hospitalizations and deaths in the aric study. Eur J Clin Nutr. (2019) 73:1133–40. doi: 10.1038/s41430-018-0346-0, PMID: 30341433 PMC6474824

[267] Kaakoush NO . Insights into the role of erysipelotrichaceae in the human host. Front Cell Infect Microbiol. (2015) 5:84. doi: 10.3389/fcimb.2015.00084, PMID: 26636046 PMC4653637

[268] He L Yang FQ Tang P Gao TH Yang CX Tan L . Regulation of the intestinal flora: a potential mechanism of natural medicines in the treatment of type 2 diabetes mellitus. BioMed Pharmacother. (2022) 151:113091. doi: 10.1016/j.biopha.2022.113091, PMID: 35576662

[269] Zhang L Zhang Q Teng D Guo M Tang K Wang Z . Fgf9 recruits beta-catenin to increase hepatic ecm synthesis and promote nash-driven hcc. Adv Sci (Weinh). (2023) 10:e2301166. doi: 10.1002/advs.202301166, PMID: 37566761 PMC10558677

