# Supplementary material for: Pathogenesis and intervention strategies for metabolic dysfunction–associated fatty liver disease from the perspective of the gut–microbiota–liver axis
Source: Front Immunol. 2026 Feb 4;17:1667180. doi: 10.3389/fimmu.2026.1667180 (PMC12913104; doi:10.3389/fimmu.2026.1667180)
Supplement: Supplementary file 1 [file Table1.doc]

Supplementary Table S1 First-mention locations are provided (section/figure) to facilitate verification.

| Abbrev. | Full term | Reader-friendly explanation | **First mention (exact location)** |
| --- | --- | --- | --- |
| NF-κB | Nuclear factor kappa B | Core transcription factor that turns on inflammatory genes (e.g., cytokines). | **Section 4.2.4** (butyrate blocks TLR4/NF-κB overactivation) |
| MAPK | Mitogen-activated protein kinase | Kinase cascade (e.g., ERK/JNK/p38) transmitting stress/inflammation signals to the nucleus. | **Section 5.1.2 Peptidoglycans** (NF-κB and MAPK activation) |
| IRF7 | Interferon regulatory factor 7 | Transcription factor that amplifies type I interferon programs in innate immunity. | **Figure 4 legend** (TLR9 suppresses IRF7 phosphorylation…) |
| GSDMD | Gasdermin D | Pore-forming effector cleaved downstream of inflammasomes; drives pyroptotic inflammatory cell death. | **“Enterobacteria–liver axis” LPS paragraph** (GSDMD-N release) |
| TAK-242 | Resatorvid | Small-molecule inhibitor of TLR4 signaling used to validate LPS–TLR4 mechanisms. | **Section 5.3.2 Endotoxemia and TLR4** (TAK-242 intervention) |
| PAMPs | Pathogen-associated molecular patterns | Conserved microbial motifs (e.g., LPS, PGN, CpG DNA) sensed by innate receptors. | **Section 5.3.2 Endotoxemia and TLR4** (“gut-derived PAMPs signaling…”) |
| MyD88 | Myeloid differentiation primary response 88 | Adaptor that relays TLR signals to NF-κB/MAPK activation. | **Section 5.3.2** (MyD88-dependent signaling axis) |
| TRIF | TIR-domain-containing adaptor inducing IFN-β | MyD88-independent adaptor downstream of TLR4; links to interferon/inflammasome pathways. | **Section 5.3.2** (TLR4 downstream TRIF pathway) |
| NLRP3 | NACHT, LRR and PYD domains-containing protein 3 | Inflammasome sensor assembling a caspase-1 complex that matures IL-1β/IL-18. | **Section 4.2.4** (NLRP3 inflammasome activation) |
| PRRs | Pattern recognition receptors | Innate immune sensors that detect PAMPs/DAMPs (e.g., TLRs, NODs). | **Section 6.1.6 Fecal transplantation** (“pattern recognition receptors”) |
