# Supplementary material for: Pathogenesis and intervention strategies for metabolic dysfunction–associated fatty liver disease from the perspective of the gut–microbiota–liver axis
Source: Front Immunol. 2026 Feb 4;17:1667180. doi: 10.3389/fimmu.2026.1667180 (PMC12913104; doi:10.3389/fimmu.2026.1667180)
Supplement: Supplementary file 2 [file Table2.docx]

Supplementary Table S2. Selected registry-listed clinical trials and translational studies targeting the gut microbiota–immune axis in MAFLD/MASH (identifiers provided for traceability; protocols/endpoints may be updated in the registry).

| **ClinicalTrials.gov ID** | **Intervention (category)** | **Population / design (brief)** | **Key immune / barrier readouts (examples)** | **Key liver endpoints (examples)** |
| --- | --- | --- | --- | --- |
| **NCT02469272** | Lean-donor FMT (microbiota replacement) | NASH / metabolic liver disease; FMT feasibility/efficacy testing | Gut permeability / endotoxin-related markers; microbiome composition | Hepatic steatosis/activity (imaging- or histology-based, per protocol) |
| **NCT03803540** | FMT (histology-focused protocol) | Biopsy-confirmed disease; includes pre/post biopsy assessment | Microbiome and inflammation-related profiling (per protocol) | **Histologic response/resolution defined on liver biopsy** (pre vs follow-up) |
| **NCT02496390** | Allogenic vs autologous FMT (randomized) | Metabolic syndrome + NAFLD; RCT | **Small intestinal permeability** (e.g., lactulose:mannitol), inflammation markers; microbiome | **HOMA-IR/insulin sensitivity**, **hepatic fat by MRI-PDFF**, metabolic outcomes |
| **NCT05607745** | Dietary counseling + lean vegan-donor FMT | NAFLD; microbiota/diet coupling | Microbiota composition; metabolite signatures (e.g., SCFAs/bile acids, per protocol) | Liver fat/activity measures and metabolic indices (per protocol) |
| **NCT04465032** | Consecutive FMT (repeated dosing) | NAFLD; repeated FMT understood as “ecology remodeling” | Microbiome shifts; permeability/endotoxemia-related markers (per protocol) | Liver fat and metabolic endpoints (protocol-defined) |
| **NCT05821010** | Synbiotics ± FMT capsules (combined strategy) | Fibrotic NASH; mechanistic translational trial | Microbiome function, inflammatory/immune readouts; metabolomics (protocol-driven) | Liver activity/fibrosis endpoints and metabolic readouts (protocol-defined) |
| **NCT06873165** | Pasteurized Akkermansia muciniphila (next-gen microbial therapeutic) | Metabolic liver disease/metabolic dysfunction; safety/efficacy | Immune/metabolic biomarkers (per protocol) | Liver biochemistry / steatosis-related endpoints (per protocol) |
| **NCT06537882** | Synbiotics (often combined with standard metabolic supplements) | MAFLD; interventional trial | Inflammation and gut-related biomarkers (per protocol) | Liver enzymes, non-invasive steatosis/fibrosis indices (per protocol) |
| **NCT05885373** | SIM01 (microbiome modulation) | Female NAFLD; single-arm | Microbiome-related profiling (per protocol) | **Liver biochemistry** (ALT/AST etc.) as primary clinical readout |
| **NCT03467282** | Probiotic supplementation | NASH; gut microbiota modulation focus | Microbiota composition; inflammatory markers (per protocol) | **Hepatic fibrosis/steatosis measures** (non-invasive and/or protocol-defined) |

Note: ClinicalTrials.gov identifiers are provided for traceability. Trial status, protocol details, and endpoints may be updated; readers are encouraged to verify the latest information in the registry. Where available, key publications are cited; otherwise, “—” indicates no primary results publication identified at the time of writing
